# Degradation of Phytate by the 6-Phytase from *Hafnia alvei*: A Combined Structural and Solution Study

**DOI:** 10.1371/journal.pone.0065062

**Published:** 2013-05-31

**Authors:** Antonio Ariza, Olga V. Moroz, Elena V. Blagova, Johan P. Turkenburg, Jitka Waterman, Shirley M. Roberts, Jesper Vind, Carsten Sjøholm, Søren F. Lassen, Leonardo De Maria, Vibe Glitsoe, Lars K. Skov, Keith S. Wilson

**Affiliations:** 1 Structural Biology Laboratory, Department of Chemistry, University of York, York, United Kingdom; 2 Novozymes A/S, Bagsværd, Denmark; University of Hong Kong, Hong Kong

## Abstract

Phytases hydrolyse phytate (*myo*-inositol hexakisphosphate), the principal form of phosphate stored in plant seeds to produce phosphate and lower phosphorylated *myo*-inositols. They are used extensively in the feed industry, and have been characterised biochemically and structurally with a number of structures in the PDB. They are divided into four distinct families: histidine acid phosphatases (HAP), β-propeller phytases, cysteine phosphatases and purple acid phosphatases and also split into three enzyme classes, the 3-, 5- and 6-phytases, depending on the position of the first phosphate in the inositol ring to be removed. We report identification, cloning, purification and 3D structures of 6-phytases from two bacteria, *Hafnia alvei* and *Yersinia kristensenii*, together with their pH optima, thermal stability, and degradation profiles for phytate. An important result is the structure of the *H. alvei* enzyme in complex with the substrate analogue *myo*-inositol hexakissulphate. In contrast to the only previous structure of a ligand-bound 6-phytase, where the 3-phosphate was unexpectedly in the catalytic site, in the *H. alvei* complex the expected scissile 6-phosphate (sulphate in the inhibitor) is placed in the catalytic site.

## Introduction

Plants use phytate (*myo*-inositol hexakisphosphate here called InsP_6_; Ins = inositol = cyclohexane-1,2,3,4,5,6-hexol) as their main storage form of phosphorous, inositol and a variety of minerals and it accounts for 75–80% of the total phosphorous in seeds such as those of cereals and legumes [1], [2]. Apart from sequestering phosphorous, phytate has additional anti-nutritional properties as it can form insoluble complexes with proteins and nutritionally important minerals such as magnesium, zinc, iron and calcium [3]. Phytases (*myo*-inositol hexa*kis*phosphate phosphohydrolases) catalyse the hydrolysis of phytate into inorganic phosphate (P_i_) and lower phosphorylated *myo*-inositols. While phytases have been isolated from a variety of microorganisms, plants and some animal tissues [4], [5], [6], monogastric species including poultry and pigs as well as humans lack the ability to produce phytate degrading enzymes or simply produce them in insufficient amounts to enable direct use of phytate from the food chain [7]. Over the past two decades the economic value of phytases has increased significantly reflecting their increasingly standard use as animal feed supplements to release the phosphorous locked up in phytate and make it bioavailable [8]. The addition of phytase reduces the need to supplement the feed with P_i_, a costly non-renewable resource that is estimated to be depleted within 50 years [9] [10]. Furthermore, it lessens the anti-nutritional effects and decreases the environmental burden from phosphate pollution in areas of intense animal farming by decreasing the amount of phosphate excreted by animals.

**Figure 1:**
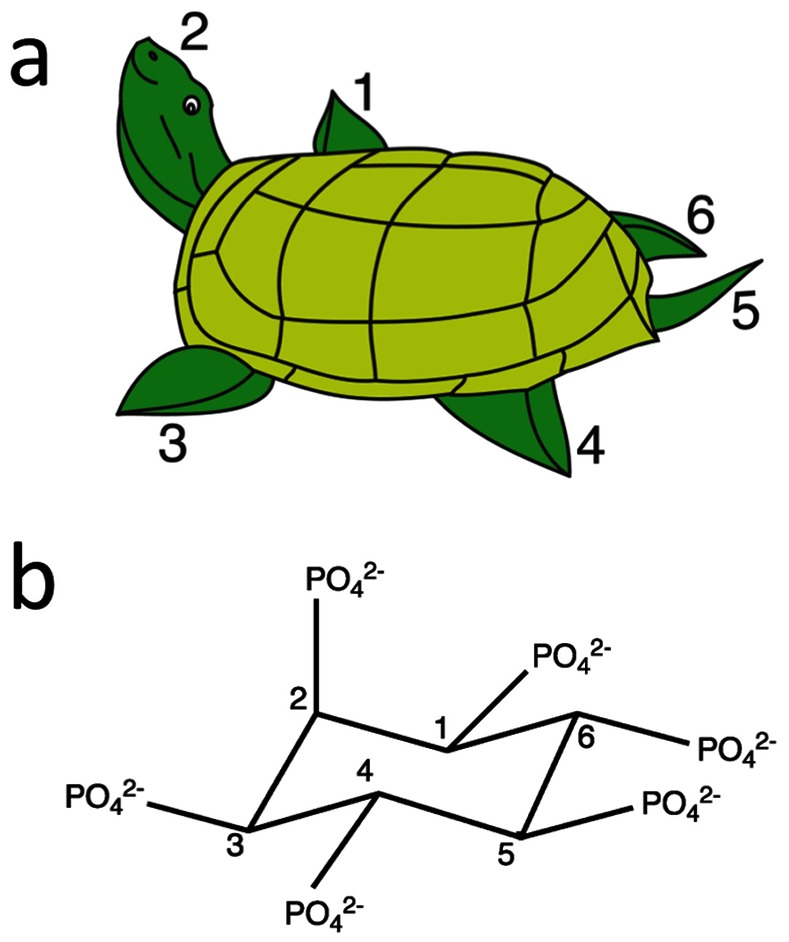
Agranoff’s rule for inositol numbering. (**a**) Agranoff’s turtle with the head up in the 2-phosphate position. (**b**) Phytate numbered according to Agranoff’s nomenclature, with the 2-phosphate axial and pointing upwards, with the carbon atoms numbered anticlockwise around the ring.

Phytic acid (the protonated form of InsP_6_) is a symmetric molecule with six dihydrogenphosphate substituents on the *myo*-inositol core. Titration experiments indicate that phytic acid can carry up to twelve protons, six with pK_a_ ∼2.2, two ∼5.7 and four ∼ 9.2 [11], [12]. It is thus likely to carry four protons at pH 7 (the main species being InsP_6_^8−^) and six at pH 5 (the main species being InsP_6_^6−^). In solution the most stable conformation of the cyclohexane ring of InsP_6_ is a ^4^C_1_ chair, with five of the six phosphates in the equatorial orientation, while the sixth (on carbon C_2_) is axial. We adhere throughout to the convention recommended by the Nomenclature Committee of the International Union of Biochemistry [13] based on the mnemonic rule first proposed by Bernard Agranoff, a pioneer of inositol chemistry [14], and used in most later studies [15], [16]. *Myo*-inositol (or here its hexakisphosphate derivative) is depicted as a turtle with the head (axial phosphate) pointing upward. The 1D numbering starts with the turtle's right flipper (position 1) and goes anticlockwise (Fig. 1a,b) (the 1L alternative has the numbering in the opposing direction).

**Figure 2:**
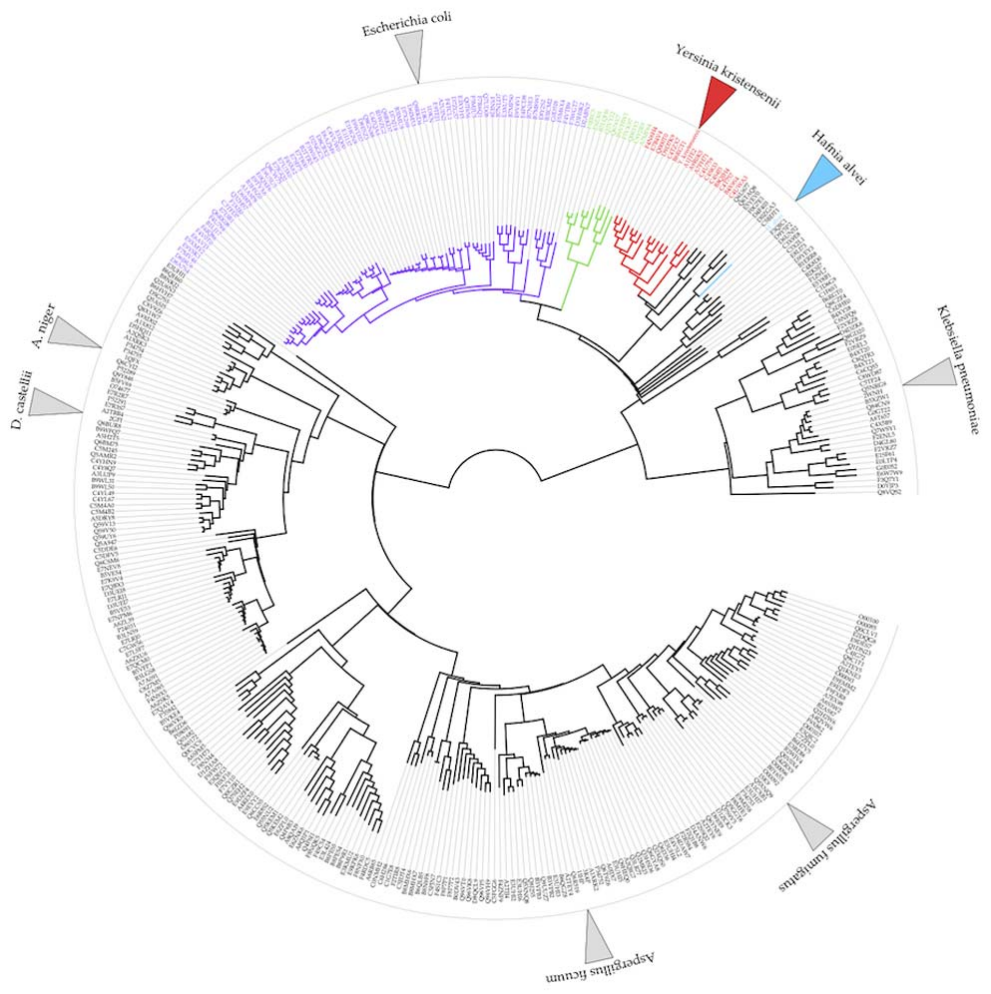
Wheel of the HAP phytases. The branches of *Escherichia* (purple), *Citrobacter* (light green) and *Yersinia* (red) are colour coded. The wheel represents a neighbour-joining of pairwise Smith-Waterman alignment scores. A full list of the UniProt entries can be found in the Supporting Information S1. The grey triangles indicate PDB entries, while the red triangle is *Yk*Phy and the blue triangle is *Ha*Phy.

**Figure 3:**
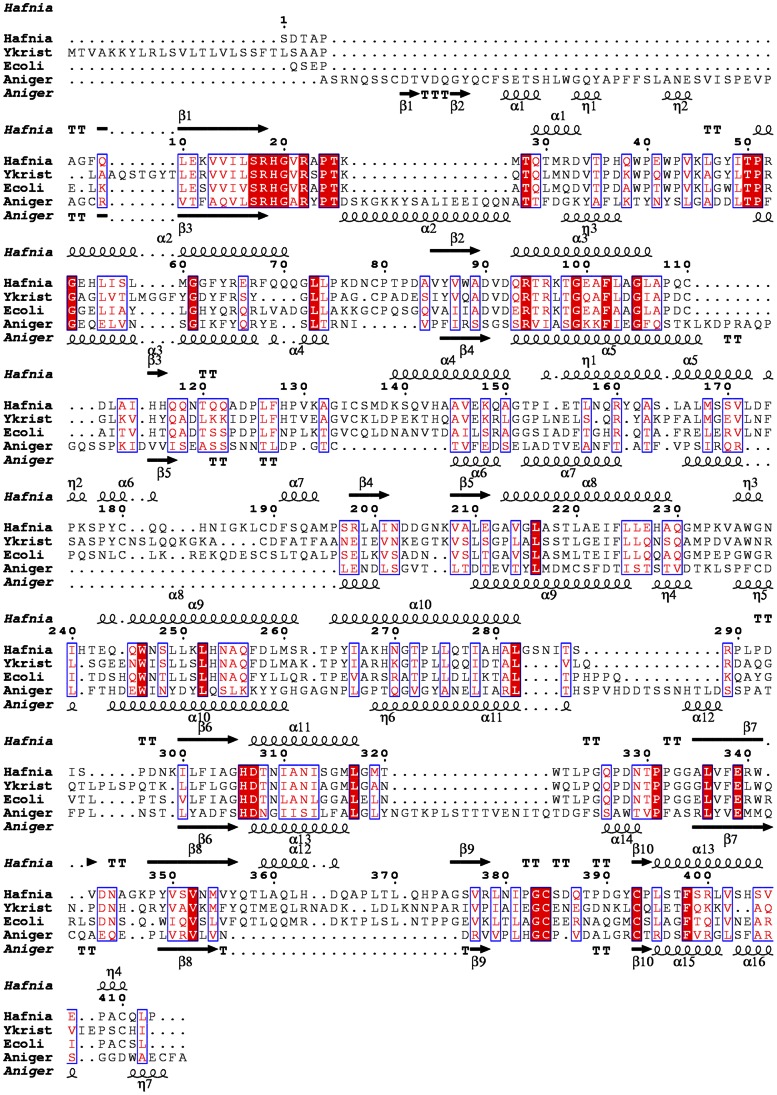
Structure-based sequence alignment for four representative phytases: *Ha*Phy, *Yk*Phy, *Ec*Phy and *An*Phy. The alignment is based on the 3D structures of *Ha*Phy, *Ec*Phy and *An*Phy using the T-Coffee Expresso web server [67], [68] – three is the maximum number of structures allowed by the server. The secondary structure elements of *Ha*Phy are shown above, and of *An*Phy below, the alignment. Fully conserved resides are shown in red boxes. The Figure was generated using ESPript [69], [70].

The phytases are divided into four homology-based families: histidine acid phosphatases (HAP), β-propeller phytases, cysteine phosphatases and purple acid phosphatases [9]. The majority are HAPs [17], [18] and are part of a large superfamily of phosphatases [19]. Here we focus on the limited number of HAPs that show activity against phytate, henceforth referred to as HAPPs. While a large number of putative HAPP genes have been identified, only a few crystal structures have been reported (Fig. 2). These include the fungal phytases from *Aspergillus ficuum* [20], *A. fumigatus* [21], *A. niger* (*An*Phy) [22] and *Debaryomyces castellii* [23], and the bacterial phytases from *Escherichia coli* (*Ec*Phy) [24] and *Klebsiella pneumoniae* [25]. HAPPs show a typical HAP fold with a large α/β-domain and a small α-domain [20], [24], [26]. The sequences of *Hafnia alvei* phytase (*Ha*Phy), *Yersinia kristensenii phytase* (*Yk*Phy), *Ec*Phy and *An*Phy are aligned in Fig. 3– this is a structure-based alignment as detailed in the discussion and the Figure legend. The enzymes share a common catalytic site architecture located at the interface of the two domains involving the conserved N-terminal active site motif RHGXRXP and the C-terminal HD. The two-step reaction involves a nucleophilic attack on the phosphorous by the histidine from the RHGXRXP motif to form a covalent phosphohistidine intermediate while the aspartic acid of the HD motif serves as a proton donor to the oxygen atom of the scissile phosphomonoester bond [27], [28], [29]. The need for the aspartate carboxylate group to be protonated in order to donate a proton to the leaving group explains the acidic pH optimum [27].

The enzymes are additionally classified into 3-, 5- or 6-phytases (EC 3.1.3.8, EC 3.1.3.72 and EC 3.1.3.26, respectively) based on the carbon position on the inositol ring at which they initiate phosphate hydrolysis. Thus 3-phytases first remove the phosphate group at the C_3_ or C_1_ position (1L- vs. 1D-convention) while 6-phytases do so at the C_6_ position (or C_4_ in the 1L convention) [30]. Very few 5-phytases have been identified (for example that from *Selenomonas ruminantium* [31]). However, phytases sequentially remove several phosphate groups from phytate, so a simple definition of substrate specificity is not straightforward. Furthermore, even in the first step different positions can be attacked, e.g. a 6-phytase can produce both the main product Ins(1,2,3,4,5)P_5_ but in addition some Ins(1,2,4,5,6)P_5_ in the first hydrolysis step [32], where the numbers in the parentheses refer to the positions of the phosphates. Structures of phytate or phytate-analogue complexes have been reported with either the 3- or 5-phosphate group in the catalytic centre. For the 3-position these are *Ec*Phy [22] (PDB code 1dkq) and *An*Phy [24] (PDB 3k4q) from the HAPP family, and a *B. subtilis* β-propeller phytase [33] (PDB 3ams, 3amr); while for the 5-position there is a *S. ruminantium* cysteine phytase [31] (PDB 1u26).

It has become common practice to add microbial phytases to animal feed since they were first commercialized in the early 1990’s. The first commercial phytases were derived from various fungal *Aspergillus* donors, e.g. from *A. niger* in the Natuphos product (BASF, Ludwigshafen, Germany) classified as a 3-phytase. Later fungal 6-phytases originating from *Peniophora lycii* were introduced (RONOZYME P and RONOZYME NP; DSM Nutritional Products, Basel, Switzerland). RONOZYME NP is a *P. lycii* variant with improved intestinal and thermal stability. During the last ten years a number of products based on *Ec*Phy has been marketed e.g. Phyzyme XP (Danisco Animal Nutrition, Marlborough, United Kingdom), OptiPhos (JBS United, Indiana, US) and Quantum (AB Enzymes, Darmstadt, Germany), the latter being a thermostabilized variant. Recently, a 6-phytase from *Citrobacter braakii* was commercialized as RONOZYME HiPhos (DSM Nutritional Products, Basel, Switzerland) [34]. All of these commercial products are HAPPs with activity in the acidic range. The phytases from *C. braakii* and *Ec*Phy are both from enterobacterial donors and have similar pH profiles with an optimum around pH 3.5–4.5. The fungal *An*Phy and *P. lycii* phytases have pH optima around 5.5 and 4–5, respectively.

Here we report the identification of two HAPPs from the enterobacteria *Hafnia alvei* (*Ha*Phy) and *Yersinia kristensenii* (*Yk*Phy), together with biochemical studies of their pH and temperature optima, as well as their phytate degradation pattern, which show that they are 6-phytases. We present structures of both enzymes: apo-*Ha*Phy, its complexes with the phytate analogue *myo*-inositol-hexakissulphate (MIHS-*Ha*Phy) and the competitive inhibitor L(+)-tartrate (tar-*Ha*Phy) and *Yk*Phy with one of its products P_i_ (P_i_-*Yk*Phy). The two enzymes are closely related and structurally similar to *Ec*Phy. The MIHS-*Ha*Phy complex is the first structure of a 6-phytase showing a phytate analogue with the 6-position in the catalytic site.

## Experimental Procedures

### Phytase Production and Purification

In a screen for new phytases, two phytase-positive bacterial strains that produce enzymes with activity at acidic pH were isolated from a sample of wet soil collected in South Zealand, Denmark. The strains were identified as isolates of *Yersinia kristensenii* and *Hafnia alvei* from their partial 16SrDNA sequences. Details of cloning, expression, protein production and purification are given in SI.

### Determination of Phytase Activity

Activity was determined with an end-point assay measuring total released phosphate from a sodium phytate solution adapted from the method of Engelsen *et al*. [35] to the microtitre well format. In brief, 75 µl phytase solution diluted in varying amounts of 0.25 M sodium acetate pH 5.5,0.005% (w/v) Tween-20 was dispensed in a microtitre plate well (NUNC 269620) before 75 µl substrate [prepared by dissolving 100 mg sodium phytate from rice (Sigma, P0109) in 10 ml 0.25 M sodium acetate buffer pH 5.5] were added. The plate was sealed and incubated for 15 min at 37°C while being shaken at 750 rpm. After incubation, 75 µl stop reagent (prepared by mixing 10 ml molybdate solution [10% (w/v) ammonium hepta-molybdate in 0.25% (w/v) ammonia solution] with 10 ml ammonium vanadate (0.24% commercial product from Bie&Berntsen, Cat.No. LAB17650) and 20 ml of 21.7% (w/v) nitric acid) was added and the absorbance measured at 405nm. The phytase activity was expressed in FYT units, with one FYT being the amount of enzyme that liberates 1 µmol inorganic ortho-phosphate/min under the above conditions. An absolute value for the measured activity was obtained by reference to a standard curve made from dilutions of a phytase enzyme preparation with known activity.

### pH and Temperature Stability

The pH-dependent activity profiles were determined at 37**°**C in the pH range 2.0 to 7.5 (in 0.5 pH-unit steps) as described in Section 3.2, except that a buffer cocktail containing 50 mM glycine, 50 mM acetic acid, 50 mM Bis-Tris (pH was adjusted to the appropriate value) was used instead of the 0.25 M sodium acetate pH 5.5 buffer. The temperature profile was determined in the range 20–90**°**C in PCR tubes instead of microtitre plates. After a 15 min reaction period at the desired temperature the tubes were cooled to 20**°**C for 20 sec and 150 µl of each reaction mixture was transferred to a microtitre plate. 75 µl stop reagent was added and the absorbance at 405 nm was measured.

### Differential Scanning Calorimetry

An aliquot of purified phytase was dialysed against 2× 500 ml 20 mM sodium acetate pH 4.0 at 4°C in a 2–3 h step followed by an overnight step. The sample was filtered (0.45 µm) and diluted with buffer to approximately 2 A_280nm_ units. The dialysis buffer was used as reference in Differential Scanning Calorimetry (DSC). The samples were degassed using vacuum suction and stirring for approximately 10 min. A scan was performed on a MicroCal VP-DSC at a constant scan rate of 1.5°C/min from 20–90°C. The MicroCal Origin software (version 4.10) was used to estimate the denaturation temperature T_d_ (melting temperature, T_m_).

### *In Vitro* Activity of HaPhy and YkPhy

The effect of the two phytases on the degradation of phytate was evaluated in an *in vitro* system mimicking the passage through the stomach. Incubation was conducted first at 40°C and pH 3.0 (60 min) followed by pH 4.0 (30 min) in the presence of pepsin (3000 U/g feed) and 0.8 g of a model feed consisting of 30% soybean meal and 70% corn with approximately 7 g Ca^2+^/kg feed, while dosing the phytases at 125 and 250 FYT/kg of feed. Following incubation, the inositol phosphates (**InsP**) were extracted from the *in vitro* samples following a slight modification of the method of Carlsson *et al*. [36], (Fig. S2). In brief, 5.6 ml of 1 M HCl were added to each sample (5.6 ml) and the resulting samples were mixed intermittently at 500 rpm for 3 h at 40°C interrupted by a freezing step. Subsequently, the samples were centrifuged (1,800×g, 4°C, 5 min), the supernatants were recovered and filtered by centrifugation (11,000×g, 0°C, 60 min) using ultracentrifugal filter devices (Microcon YM-30, Millipore, Billerica, MA) prior to analysis by High Performance Ion Chromatography.

### Analysis of Inositol Phosphates by HPIC

InsP_6_-InsP_3_ in samples from the *in vitro* and degradation pathway studies were analysed using High Performance Ion Chromatography (HPIC; Dionex Corp., Sunnyvale, CA) as described in [37]. InsP_6_-InsP_3_ were detected after elution from the column by reaction with 0.1% Fe(NO_3_)_3_·9H_2_O in a 20 ml/l solution of HClO_4_, by UV absorbance at 290 nm. A reference sample for the identification of peaks was prepared by dissolving 0.5 g of phytic acid dodecasodium salt hydrate in 50 ml of 0.5 M HCl and autoclaving the solution for 1 h at 124°C. Peaks were quantified according to an InsP_6_ standard curve (data not shown). The detector response factors of the lower inositol phosphates are lower than those of InsP_6_ and therefore the amounts of InsP_5_, InsP_4_ and InsP_3_ were estimated using the theoretical correction factors of 1.2 ( = 6/5), 1.5 ( = 6/4), and 2 ( = 6/3).

### InsP_6_ Degradation Pathway

**Figure 4:**
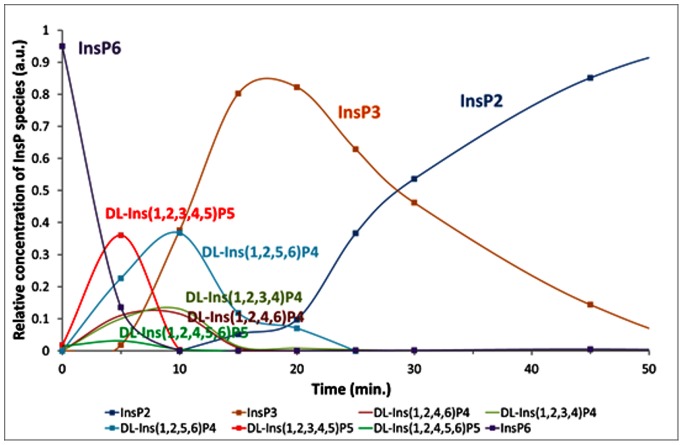
The digestion profile of phytate (InsP) by *H. alvei* phytase. The InsP profile at pH 4.0 is shown after incubation with *Ha*Phy at 37°C for 0, 5, 10, 15, 20, 25, 30, and 45 min (1 FYT/ml, 5 mM Na-phytate).

InsP_6_ degradation products were analysed using an established approach [38], [39] previously used for a number of other phytases [40], [41], [42], [43], [44], [45]. In brief, InsP_6_ and degradation products (InsP_5_-InsP_2_) generated by *Ec*Phy, *Ha*Phy and *Yk*Phy were identified by High Performance Ion Chromatography (Fig. S3) and the degradation pathways deduced (Fig. S4). Degradation was performed at pH 4.0 and pH 5.5 (0.25 M sodium acetate) by mixing 100 µl sodium phytate (10 mM) with 100 µl enzyme sample (0.5 FYT/ml) and incubating at 37°C (thermomixer 1000 rpm). The reaction was stopped at various time points (0–150 min) by addition of 200 µl 1.0 M HCl. The patterns at pH 4.0 and 5.5 were comparable and therefore only data from pH 4.0 are shown (Fig. 4).

### Structure Solution

#### Crystallisation and data collection

Crystallisation was performed at room temperature with commercially available screens using sitting-drop vapour-diffusion. Drops were set up employing a *Mosquito Crystal* liquid handling robot (TTP LabTeck, UK) with 150 nl protein solution plus 150 nl reservoir solution in 96-well format plates (MRC 2-well crystallisation microplate, Swissci, Switzerland) equilibrated against 54 µl reservoir solution.

#### Apo-*Ha*Phy

A crystal cluster formed in 0.1 M HEPES pH 7.5, 10% isopropanol, 20% (w/v) PEG 4000 using protein at a concentration of 7.7 mg/ml. Individual crystals were separated from the cluster and cryoprotected by dipping them into a solution containing 25% glycerol for 5 sec before vitrifying in liquid nitrogen. X-ray data were collected using a Rigaku Micromax-007 X-ray generator (Cu *K*α, λ = 1.54179 Å) equipped with a MAR345 image plate detector (Marresearch GmbH, Germany) to a resolution of 1.90 Å in space group *P*6_3_22.

#### Tar-*Ha*Phy: tartrate complex

In a first attempt to obtain a complex with myo-inositol hexakis-sulphate (MIHS), the protein at a concentration of 7.7 mg/ml containing MIHS at a 3∶1 molar ratio of inhibitor to protein was incubated at 4°C overnight. Crystals formed in condition H2 of the Index screen (Hampton Research, USA): 0.2 M potassium/sodium tartrate +20% (w/v) PEG 3350, pH 8.4–9.1 and were vitrified without the addition of a cryoprotectant. X-ray data were recorded at beam line ID23–1 of the European Synchrotron Radiation Facility (ESRF, Grenoble) at a wavelength of 1.0723 Å to a resolution of 1.60 Å and were in space group *C*222_1_. MIHS was subsequently shown not to be bound.

#### MIHS-*Ha*Phy complex

MIHS binding to *Ha*Phy was characterised by isothermal titration calorimetry (MicroCal VP-ITC). The protein was dialysed against 50 mM sodium acetate pH 4.5 and the ligand was dissolved in the same buffer. The protein concentration in the cell was 0.055 mM and the ligand concentration in the syringe 0.55 mM, with 18 injections at room temperature. The optimal pH for *Ha*Phy activity is ∼4.5 therefore sodium acetate buffer pH 4.5 was used for the titration experiment. MIHS (Sigma) was diluted in sodium acetate buffer pH 4.5 to 20 mM concentration and mixed with the protein to reach a final concentration of 5 mM. Crystals grew in condition D12 of the JCSG screen (Molecular Dimensions Ltd): 0.04 M KH_2_PO_4_, 16% (w/v) PEG, 20% (v/v) glycerol. Data were collected at beam line I04 of the Diamond Light Source (Didcot, UK) at a wavelength of 0.9795 Å to a resolution of 1.60 Å and were in space group *P*3_2_21.

#### Pi-YkPhy

Crystallisation screening was performed using protein at a concentration of 10.8 mg/ml. Large crystals were obtained in condition A2 of the PACT Screen (Molecular Dimensions, UK): SPGS (succinate, phosphate, glycine system) buffer pH 5.0, 25% PEG 1500, and were cryoprotected by dipping into a solution containing 20% glycerol for 5 sec and vitrified. Data were collected using a Rigaku Micromax-007 X-ray generator (Cu *K*α, λ = 1.54179 Å) equipped with a MAR345 image plate detector to a resolution of 1.67 Å.

#### Data processing, structure solution and refinement

X-ray data were processed using programs from the *CCP4* suite [46]. The images were integrated with *MOSFLM* [47] and scaled with *SCALA* [48], [49]. Molecular replacement (MR) solutions were obtained using *MOLREP* [50] with models derived using *CHAINSAW* [51]. The structure of apo-*Ha*Phy was solved using that of apo-*Ec*Phy (pdb code: 1dkl; which has 49% amino acid sequence identity) as a search model. The MIHS-*Ha*Phy, tar-*Ha*Phy and P_i_-*Yk*Phy structures were all solved using the refined apo-*Ha*Phy model. *Yk*Phy was rebuilt using *BUCCANEER* [52]. The structures were completed using iterative cycles of *COOT* [53] and *REFMAC5* [54]. For the MIHS-*Ha*Phy, tar-*Ha*Phy and P_i_-*Yk*Phy complexes, the refinement included Translation Libration Screw-motion (TLS) [55]. In the later stages, the contribution of the hydrogen atoms to the structure factors was taken into account. The final models showed good stereochemistry when analysed with *SFCHECK* [56] and *RAMPAGE* [57]. For the MIHS-*Ha*Phy complex, validation was performed using SFCHECK, PROCHECK [58] and MOLPROBITY [59]. Processing and refinement statistics are given in Table 1.

**Table 1:** Crystallographic statistics.

Data set	*H. alvei*: apo	*H alvei+MIHS*	*H. alvei*+tartrate	*Y. kristensenii+*P_i_
Wavelength (Å)	1.54179	0.9795	1.0723	1.54174
Space group	*C*222_1_	P3_2_21	*C*222_1_	*P*1
Cell parameters				
a (Å)	100.54	82.3	101.05	55.41
b (Å)	101.28	82.3	101.40	67.72
c (Å)	85.27	103.6	84.95	73.16
α (°)	90.00	90.00	90.00	76.67
β (°)	90.00	90.00	90.00	78.32
γ (°)	90.00	120.00	90.00	87.35
Total reflections	178749 (25302)	584723 (83875)	797655 (98005)	612939 (67373)
Unique reflections	33031 (4655)	54663 (7875)	57847 (8355)	108862 (13860)
Resolution (Å)	1.90 (2.00–1.90)	1.59 (1.68–1.59)	1.60 (1.69–1.60)	1.66 (1.75–1.66)
*R_merge_* *	7.2 (55.0)	10.4 (12.3)	10.8 (52.3)	5.0 (17.6)
Completeness (%)	95.6 (93.2)	100 (100)	100 (100)	90.8 (79.1)
Redundancy	5.4 (5.4)	10.7 (10.7)	13.8 (11.7)	5.6 (4.9)
*I/σ(I)*	13.1 (2.5)	17.2 (3.9)	17.4 (5.0)	24.9 (7.4)
V_M_ (Å^3^/Da)	2.47	2.25	2.48	2.87
Mol. per AU	1	1	1	2
R_cryst_ **	18.3	15.6	16.2	16.4
R_free_	24.1	19.2	18.9	19.9
Reflections working set	31326	51814	54887	103327
Free R-value set (no reflections)	5.2% (1702)	5.1%(2642)	5.1% (2934)	5.1% (5512)
No. of non-hydrogen atoms	3265 (pro)	3184 (pro)	3273 (pro)	6486 (pro)
No. of water molecules	303	385	643	1159
Mean B value for protein atoms (Å^2^)	29.6	25.5	12.6	17.1
Mean B value for waters (Å^2^)	39.2	36.7	24.4	24.5
B value for ligands (Å^2^)	N/A	23.3 (MIHS-1) 19.5 (MIHS-2)	11.5 (tartrate) 9.6 (tartrate)	N/A
RMS deviation from ideality				
Bonds (Å)	0.0193	0.0182	0.0140	0.0168
Angles (°)	1.529	1.87	1.631	1.805
Ramachandran statistics (%)				
Preferred region	98.1	98.3	98.6	97.6
Allowed region	1.6	1.7	1.4	2.4
Outliers	0.3^a)^	0.0	0.0	0.0

* ) R_merge_ (%) is defined as 100× Σ|I–<I>|/Σ I, where I is the intensity of the reflection.

** ) R_cryst_ = Σ||Fo|–|Fc| |/Σ|Fo| where Fo and Fc are observed and calculated structure factors respectively.

a) Asp77, belongs to a flexible loop.

#### PDB accession codes

Coordinates and structure factors have been deposited in the PDB with accession codes 4ars, 4aro, 4aru, 4arv, (r4arssf, r4arosf, r4arusf, r4arvsf for structure factors) for apo-*Ha*Phy, MIHS-*Ha*Phy, tar-*Ha*Phy and P_i_-*Yk*Phy respectively.

## Results

### Protein Preparation and Biochemical Properties

*Ha*Phy, the T308A *Ha*Phy mutant and *Yk*Phy were successfully overexpressed and purified. The pH profiles showed optimal activity of the wild type enzymes at pH 4.0–4.5 (Fig. S1a). The T308A *Ha*Phy mutant possessed only 5% of the specific activity of the wild type and it is therefore not included in Fig. S1a. The activity of *Ha*Phy at pH 7 or above is low, which explains our inability to obtain a crystal complex of *Ha*Phy with MIHS at this pH. Crystallisation of the complex was achieved when the pH of the screens was changed to ∼4.5. The activity was measured as a function of temperature at pH 5.5 (Fig. S1b) with maximum activity at 65°C for *Ha*Phy and 55°C for *Yk*Phy. The increase in activity with temperature, until unfolding occurs, is often seen for hydrolytic enzymes and is in keeping with measurements using DSC, which showed melting temperatures of 70°C for *Ha*Phy and 57°C for *Yk*Phy at pH 4.0 (data not shown).

### InsP_6_ Degradation

The three phytases reported here followed the same overall pattern, with the major InsP_6_ degradation product being DL-Ins(1,2,3,4,5)P_5_ confirming their designation as 6-phytases (Figs. 4 and S4). However, some Ins(1,2,4,5,6)P_5_, the degradation product characteristic for 3-phytases, was also detected after incubation with all three enzymes, 25% of the InsP_5_ degradation products for *Ec*Phy being Ins(1,2,4,5,6)P_5_ whereas the corresponding values for *Ha*Phy and *Yk*Phy were 11–14%. Interestingly, only one InsP_4_ degradation product was identified for *Ec*Phy (DL-Ins(2,3,4,5)P_4_). While this was the main InsP_4_ degradation product for the other two enzymes, in the incubations with *Ha*Phy and *Yk*Phy significant amounts of DL-Ins(1,2,3,4)P_4_ and Ins(1,2,4,6)P_4_ were also present. Due to the symmetry of the myo-inositol core it is impossible to distinguish certain isomers from one another, e.g. DL-Ins(2,3,4,5)P_4_ and DL-Ins(1,2,5,6)P_4_. In addition, the samples analysed only provide snapshots of the degradation pathway – other isomers could theoretically be produced and degraded between sampling.

When *Ha*Phy and *Yk*Phy were incubated in an *in vitro* system mimicking the passage through the stomach in a monogastric animal using corn and soybean meal as a substrate, there was a clear dose-dependent effect for both enzymes, with *Ha*Phy degrading phytate to a greater extent than *Yk*Phy (Fig. S2). *Ha*Phy appeared to degrade InsP_6_ and InsP_5_ at a faster rate than InsP_4_, as the InsP_4_ accumulated in the degradation process.

### The Structure of apo-*Ha*Phy

**Figure 5:**
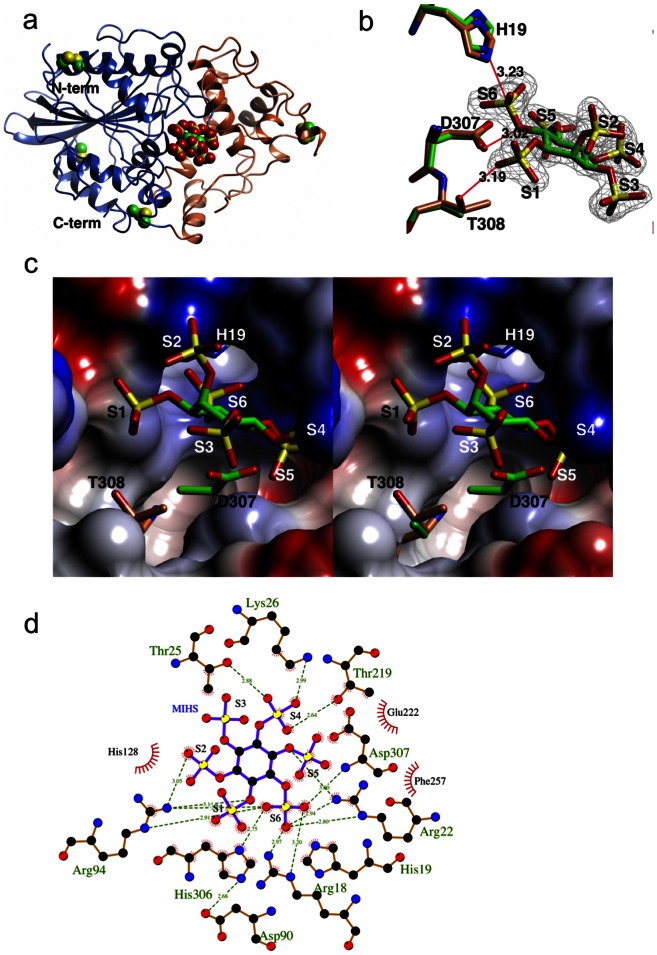
MIHS-*Ha*Phy complex structure. (**a**) Ribbon representation of the *Ha*Phy fold. The α domain (residues 25–45 and 137–264) is shown in coral, the α/β domain is in blue. The four disulphide bridges are in sphere format and lie in surface loops. The active site lies at the interface between the two domains – viz. the position of the active site bound ligand from the MIHS-*Ha*Phy complex. (**b**) Mono view of the binding of MIHS to the active site of *Ha*Phy, with the density contoured at the 1σ level for the MIHS. The six sulphates are labelled S1–S6. The side chains of three residues (His19, Asp307 and Thr308– the latter from the wild type structure) which form key interactions are shown as cylinders, with the carbons atoms coloured green for the complex structure, and coral for the wild type. The extensive additional interactions of the MIHS with the protein and water molecules are not shown for clarity. (**c**) Stereo view of the protein surface around the ligand labelled as in (b). (**d**) Schematic representation of the interactions in the active site. (a–c) were drawn using CCP4mg [71], and (d) using LigPlot+ [72].

The apo*Ha*Phy structure at pH 7.5 was refined at a resolution of 1.90 Å (Table 1) with one monomer in the asymmetric unit. The first two residues of the N-terminus, five residues (184–188) in a small disordered loop and three residues (414–416) at the C-terminus were disordered. Six side chains on the surface were modelled as dual conformers, while Glu181, Gln182 and His183 have poorly defined side chains and lie at the start of the disordered loop. The model includes seven glycerol, five acetate and 306 water molecules. The overall fold conforms to that of known HAPs with an α domain (residues 25–45 and 137–264) and a α/β domain (the remaining residues) with two α helices on each side of a twisted β-sheet (Fig. 5a). The two central helices of the α domain form part of the active site, which lies in a pocket at the interface of the two domains. The conserved N-terminal RHGXRXP and C-terminal HD motifs compose the catalytic site [26]. *Ha*Phy possesses four disulphide bridges involving cysteines 79/110, 135/410, 180/189, and 384/393, the latter being highly conserved in all known HAP structures [25], [60].

### The Structure of the Tar-*Ha*Phy and MIHS-*Ha*Phy Complexes

**Figure 6:**
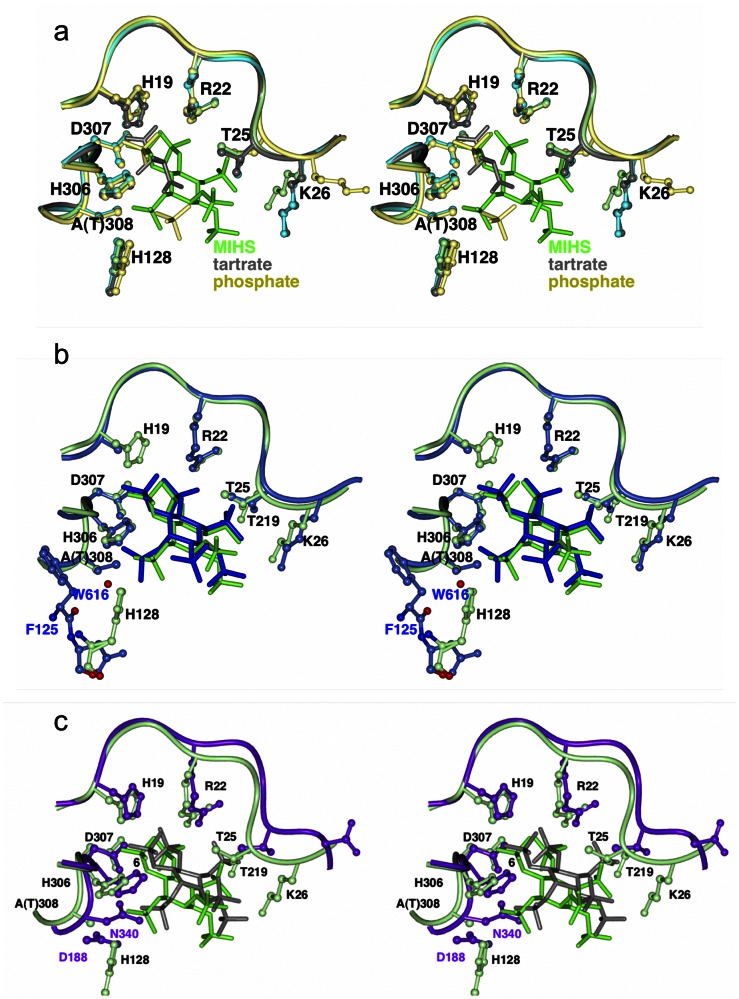
Stereo view of binding site comparisons for the HAPP phytases. (**a**) Superposition of the binding site of the MIHS*-Ha*Phy complex (green) on the corresponding region of tartrate bound *Ha*Phy (grey) and phosphate-bound *Yk*Phy (yellow). (**b**) Superposition of the binding site of the MIHS-*Ha*Phy complex (green) on the corresponding region of the *Ec*Phy phytate complex (blue). (**c**) Superposition of the binding site of the MIHS*-Ha*Phy complex on the corresponding region of the MIHS-*An*Phy complex (protein in purple, MIHS grey). The ligand-binding residues are shown in ball and stick.

The first attempt to co-crystallise *Ha*Phy with the substrate analogue MIHS [61], at pH 8.4–9.1 led to the tar-*Ha*Phy crystal. The structure was isomorphous to that of the apo-enzyme with the same disordered residues. The active site contained a tartrate molecule, a component of the crystallisation buffer and a well-known inhibitor of HAPs [62], [63]. The tartrate superimposes rather closely with the 6-sulphate moiety in the MIHS*-Ha*Phy complex and the P_i_ in the P_i_-*Yk*Phy complex described below (Fig. 6a). There is a second tartrate, modelled with an occupancy of 0.25, close to a two-fold rotation axis, where it coordinates with His116 and Gln118 from two symmetry related *Ha*Phy molecules.

Perhaps it was not surprising that MIHS failed to bind at this high pH, since the optimal pH for *Ha*Phy activity is ∼4.5 (see 4.1). Subsequently, isothermal titration calorimetry was used to confirm that MIHS bound to *Ha*Phy at pH 4.5 and allowed estimation of the K_d_ as ∼160 nM assuming a one-site model (data not shown), although the analysis was complicated by what appeared to be a degree of non-specific binding of additional MIHS. For the next screen, MIHS (Sigma I6005) was diluted in sodium acetate buffer pH 4.5 to 20 mM concentration and mixed with the protein to reach a final concentration of 5 mM. Co-crystals with the wild-type *Ha*Phy grew in clusters of insufficient quality for data collection, but a successful hit was obtained for the 5% active T308A mutant of the enzyme. While the mutant MIHS-*Ha*Phy complex crystallised in a different space group to the apo-enzyme and tar-*Ha*Phy complex, the overall fold is essentially unchanged, with one molecule in the asymmetric unit and residues 184–189 disordered. There is a difference in conformation of the first few N-terminal residues, reflecting differences in crystal packing. In the complex the electron density for residues 202–204 is very poorly defined so these residues are not modelled, and the side chain of Lys207 is disordered. In the apo- and tartrate-bound structures this region is better defined, with only the side chains of Asn206 and Lys207 disordered in both. Flexibility in this region, located at the outer border of the binding pocket, may facilitate the entry of the substrate into the active site.

#### The active site

The electron density for MIHS is very well defined, Fig. 5b, with the 6-sulphate in the catalytic centre, unambiguously confirming that this is a 6-phytase, in agreement with the degradation profile. MIHS is coordinated by Arg18, His19, Arg22, Thr25, Lys26, His128, Thr219, His306, Asp307 and a number of water molecules. The Nε_2_ atom of the catalytic nucleophile, His19, lies 3.2 Å from S6 of the MIHS and is in-line with the S-O bond from the inositol ring. The OD1 atom of Asp307, the proton donor, forms an H-bond with the oxygen at position 6 of the inositol ring, Fig. 5b,c,d. The need for the Asp307 to be protonated explains the low pH optimum for the enzyme. The activity may also be affected by the protonation state of the phytate itself, expected to carry four protons at pH 7.5, but six at pH 5 giving an ion with six negative charges [11]. The partly inactive T308A mutant retains 5% of the activity of the wild type in keeping with the loss of a single H-bond. However, the position of the Thr308 Cβ is essentially identical in the apo-wild-type, and it can confidently be assumed that the position of the ligand is closely similar in both.

#### Additional ligand binding site

There is a second MIHS bound between three symmetry-related phytase molecules in the crystal, in a completely different conformation, with the 2-sulphate in the equatorial orientation and all five others axial (Fig. S5a). It is coordinated by Gln23 and Arg52 from one protomer, Lys45 from a second and Lys347 from the third. A key feature is the presence of a potassium ion, coordinated by four oxygens from three sulphates of the MIHS (1, 3 and 5) with its ligand shell completed by the main chain oxygens of Gly230 and Gly231 (Fig. S5b). A previous phytate structure, with MIHS occupying a secondary, non-functional site, bound between two crystallographically-independent subunits was reported for the 5-phytase from *S. ruminantium* [31] (PDB 1u26) in which both MIHS ligands have one equatorial and five axial sulphates. This confirms the tendency of negatively-charged phytate to bind non-specifically to proteins and to coordinate metals, stressing again the importance of phytases in the animal feed industry to allow its hydrolysis. In the structure of sodium phytate itself [64] the phytate is in a similar conformation with five axial phosphate moieties, and only the sixth, the 2-phosphate, equatorial. These authors suggested that dipolar and coulombic repulsions between the phosphates were the dominant factor in stabilizing this conformer.

Taken together, these structures suggest that the energy difference between the five-equatorial/one-axial conformation and its inverse is not too great and that the population of the two states is strongly influenced by the local environment. This may well be significant in the sequential removal of the various phosphates from the myoinositol-hexakis-phosphate. However, we note that a predominantly axial MIHS would not fit into the *Ha*Phy active site.

### The Structure of the P_i_-*Yk*Phy Complex

The structure of P_i_-*Yk*Phy was refined at a resolution of 1.67 Å (Table 1) with two protein monomers in the asymmetric unit, each modelled with residues 6 to 414, and with very similar folds (rmsd = 0.22 Å). The overall fold is essentially identical to that of apo-*Ha*Phy (rmsd = 0.96 Å over 391 residues) and is shown in Fig. S6 in the same orientation as for *Ha*Phy in Fig. 5(a). Each monomer contains two P_i_ molecules in its active site (Fig. 6a). The first Pi superposes almost perfectly with the 6-sulphate from the *Ha*Phy-MIHS complex. The second is coordinated by His126 (corresponding to His128 in *Ha*Phy) similar to the 1-sulphate of MIHS, but does not superimpose with any of sulphates of the MIHS ring (Fig. 6b).

## Discussion

We have characterised two bacterial HAPPs, investigated their activity and phytate degradation profiles and determined the structure of a phytate analogue complex. The biochemical properties are similar and they can both be classified as predominantly 6-phytases, with *Ha*Phy showing a considerably higher thermostability. The structure of *Yk*Phy is similar to that of *Ha*Phy and so discussion focuses on the latter.

### Substrate Preference in the HAPP Active Site

The HAPPs can belong to either the 3-phytase (EC 3.1.3.8) or 6-phytase (EC 3.1.3.26) class, which share similar folds and high identity of the active site residues. There are structures of ligand complexes in the PDB for two α-α/β fold HAPPs, an inactive mutant of the *E. coli* enzyme (*Ec*Phy) in complex with phytate itself [24] and the fungal *A. niger* enzyme (*An*Phy) in complex with MIHS [22]. The sequence identities and rms differences in Cα positions in comparison to *Ha*Phy are *Yk*Phy (52.4%, 1.01Å for 394 Cα), *Ec*Phy (48.2%, 1.18Å for 391) and *An*Phy (21.6%, 2.30Å for 236) and a structure-based sequence alignment is shown in Fig. 3. The main difference in the MIHS-*Ha*Phy complex is in the position of the scissile moiety in the inositol ring. In both *Ec*Phy and *An*Phy the scissile phosphate lies in position 3, with the implication that phosphorolysis starts at this position of the ring. The presence of the 3-phytate phosphate in the catalytic site of *An*Phy, which is in accordance with its classification as a 3-phytase, was explained by the architecture of the binding site, where His338 (*An*Phy numbering) is positioned so as to favour axial orientation of the adjacent sulphate. In contrast, the corresponding residue in *Ha*Phy (His306) is further away and does not prevent the equatorial orientation of the sulphate in position 1.

*Ec*Phy was classified as a 6-phytase [65], and the phytate degradation profile reported here confirms that phosphorolysis does primarily occur at this position, with a lesser activity at the 3-position. However, the published structure of the phytate complex with the inactive *Ec*Phy mutant showed the 3-phosphate bound in the scissile position [24], (PDB 1dkp). In this structure there are two alternatively occupied mercury sites positioned close to phytate, which can be presumed to cause perturbations in the ligand geometry, but it is not clear that these are sufficient to explain the unexpected placing of the 3-phosphate in the catalytic site. Why *Ec*Phy should have the 3-phosphate in the catalytic site remains unexplained and it is possible that this arises from an accumulation of small differences rather than any one single change.

However, one important difference between *Ec*Phy and *Ha*Phy is that there are much more pronounced conformational changes upon ligand binding in the former. In *Ha*Phy, ligand binding does not lead to significant conformational changes in residues 22–27 (corresponding to 20–25 in *Ec*Phy). While Arg20 in *Ec*Phy moves significantly to form a contact with the scissile phosphate, in *Ha*Phy the corresponding Arg22 is already in the “contact-ready” conformation in the apo-structure, superimposing very well on the ligand-bound arginines from both the *Ec*Phy and *Ha*Phy complexes. Similarly, there are no significant conformational changes in the next five residues, although the side chain of Lys26 does move slightly (N_Z-_apo to N_Z-_complex shift of 3.2 Å) to make a contact with the O4 of the 4-sulphate of MIHS. This is quite different from the more dramatic events in *Ec*Phy, where the main chain of the corresponding Lys24 moves by 4.7 Å, giving a large repositioning of the side-chain (N_Z-_apo to N_Z-_complex shift of 15 Å), to make a contact with the oxygen from the 6-phosphate of the ligand. This position is occupied by the 3-sulphate in the MIHS-*Ha*Phy structure. While His128 in MIHS-*Ha*Phy coordinates the 1-sulphate, in *Ec*Phy the interaction with the axial 2-phosphate (which is closest to the 1-sulphate in MIHS-*Ha*Phy) is provided by a water molecule that forms a bond with the main chain oxygen of Phe125 (Fig. 6b). In *An*Phy a similar interaction with the 2-sulphate is formed by Asp188 (Fig. 6c).

Thr219, which coordinates the 4-sulphate in MIHS-*Ha*Phy, is equivalent to Met216 in *Ec*Phy, which has no contacts with the ligand. There are no equivalent residues in *An*Phy; the 5-sulphate in this structure, which is the closest to 4-sulphate in MIHS-*Ha*Phy, is coordinated by Tyr28 and Lys68 (not shown, so as not to overload Fig. 6c). Thr308 has been mutated to alanine in the MIHS-*Ha*Phy complex, so a potential H-bond to the 1-sulphate is missing: Thr305, the equivalent residue in *Ec*Phy, coordinates the axial 2-phosphate (Fig. 6b). It is unlikely that the loss of this H-bond in *Ha*Phy is responsible for the change in phytate orientation.

Yet another difference from the *Ec*Phy active site structure is that there is no change in the conformation of Glu222 (Glu219 in *Ec*Phy) between the apo and ligand-bound states. The ligand-induced conformational changes in this residue were proposed to be important for catalysis in *Ec*Phy. However, in *An*Phy, as in *Ha*Phy, there are essentially no conformational changes, confirming that the movement of this Glu is not required for catalysis in all HAPPs. Indeed the movement of Glu219 in *Ec*Phy may be a result of changes of pH [24]. For *Ec*Phy, the apo-structures were determined at different pHs (4.5, 5.0 and 6.6) and the conformation of Glu219 in the apo-form differs from that of the phytate-bound form only at pHs 4.5 and 5.0, while at pH 6.6 Glu219 adopts the phytate-bound conformation.

The *Yk*Phy structure is very similar to that of *Ha*Phy, with a very close overlap of the active site residues, suggesting that it is also a 6-phytase in accordance with our biochemical data. One of the important residues determining the binding pocket specificity is His306, corresponding to His338 in *An*Phy, which determines the axial orientation of the adjacent phosphate. This His is in the same conformation as in *Ha*Phy, suggesting the phytate phosphates will be in the same orientation. Although the structures of both enzymes are closely similar, *Ha*Phy has a melting temperature about 13°C higher than that of *Yk*Phy, Fig. S1b. Thermostability is a key property in animal feed, where a commercial phytase that can survive feed processing, e.g. pelleting at 90°C, has a competitive advantage. However, pelleting stability is a combination of intrinsic stability and formulation, and adequate solid formulation of the final product is still a prerequisite for high survival.

### Flexibility and Specificity

Our combined solution and structural study demonstrates that *Ha*Phy and *Yk*Phy show a strong preference for the first phosphorolytic degradation step being the removal of the 6-phosphate of the phytate. This is in contrast to the *Ec*Phy complex where the 3-phosphate is in the catalytic centre, in spite of an earlier report of a preference for the 6-position [41]. In sequence comparisons, *Ec*Phy lies close to other bacterial 6-phytases (Fig. 2), and quite far from the bacterial 3-phytase from *Klebsiella* ASR1 [40], in agreement with the results of our digestion studies, One explanation for the 3-phosphate being in the catalytic site in the *Ec*Phy phytate complex could be the mutation of the catalytic histidine in that structure. However, the 6- and indeed the 3-phytases process the phytate sequentially down to the mono-inositol phosphate [66], which must require some flexibility in the conformation of the substrates as phosphorolysis proceeds. Indeed, the ability of phytate to take up alternative conformations is evidenced by the unusual structure of the second MIHS bound between three protomers in the crystal with five axial and one equatorial ligand.

In addition, we note that in our measurements 25% of the InsP6 degradation products for *Ec*Phy was Ins(1,2,4,5,6)P5 whereas the corresponding values for *Ha*Phy and *Yk*Phy were 11–14%. This means there is less specificity in the interaction of *Ec*Phy with phytate, and the likelihood of its crystallising in the 3-position was 1∶4, in contrast to HaPhy where it was closer to 1∶10. We propose, that it may be His128 (coordinating the phosphate(sulphate)-1) which determines the higher specificity for a scissile phosphate in the six-position, because the only space remaining for the axial sulphate is outside the binding pocket.

### Conclusion

Our degradation studies in solution of the phytases from *H. alvei* and *Y. kristensenii* (and indeed *E. coli*) identify them as 6-phytases with a preference for removing the 6-phosphate from phytate. The X-ray studies on *Ha*Phy and *Yk*Phy double the number of bacterial HAPPs for which structures are available which in combination with biochemical characterisation data will contribute to better understanding of the structure-function relations in this family. In the structure of the complex of *Ha*Phy with MIHS, the 6-sulphate is bound in the catalytic site as expected for an enzyme functioning as a 6-phytase, the first report of a HAPP 6-phytase complex. The presence of the second MIHS ion in the crystal packing illustrates phytate’s capability of binding proteins and minerals *in vivo*. Our high resolution structure gives a detailed picture of the interactions between the substrate and the enzyme when working as a 6-phytase. Protein engineering will now be better informed when exploiting the structural data to optimise various properties (e.g. pH-optimum, specific activity and thermal stability) that are important for industrial applications. Optimisation of these properties is key for future application of phytases since the continuing development requires more and more efficient enzymes. *Ha*Phy with its intrinsic high thermostability is an excellent candidate for such protein engineering and has the potential to progress towards a commercial product in the animal feed industry.

## Supporting Information

Figure S1 **(a) The relative activity of the two enzymes as a function of pH (b) The relative activity as a function of temperature.** In both a) and b) the values are relative % activity normalized to the value at optimum for each phytase.
(TIFF)

Figure S2 **Residual inositol phosphates (InsP_6_-InsP_3_; mg InsP-P/g feed) after** ***in vitro*** **incubation without phytase or with** ***Ha*** **Phy or** ***Yk*** **Phy dosed at 125 and 250 FYT/kg feed.**
(TIFF)

Figure S3 **HPIC analysis of the hydrolysis products of myo-inositol hexakisphosphate (InsP_6_-InsP_2_) by the purified phytase after** ***in vitro*** **incubation for 0, 5, 10, 30 and 120 min at pH 4.0.** Reference sample of hydrolysed Na-phytate. Peaks: (1) InsP_1_; (2) Phosphate; (3–4) InsP_2_; (5) Ins(1,3,5)P_3_; (6) Ins(2,4,6)P_3_; (7) DL-Ins(1,2,4)P_3_; (8) DL-Ins(1,2,6)P_3_, Ins(1,2,3)P_3_; (9) DL-Ins(1,4,5)P_3_; (10) DL-Ins(1,5,6)P_3_; (11)Ins(4,5,6)P_3_; (12) Ins(1,2,3,5)P_4_; (13) DL-Ins(1,2,4,6)P_4_; (14) DL-Ins(1,2,3,4)P_3_; (15) Ins(1,3,4,6)P_4_; (16) DL-Ins(1,2,4,5)P_4_; (17) DL-Ins(1,3,4,5)P_4_; (18) DL-Ins(1,2,5,6)P_4_; (19) Ins(2,4,5,6)P_4_; (20) DL-Ins(1,4,5,6)P_4_; (21) Ins(1,2,3,4,6)P_5_; (22) DL-Ins(1,2,3,4,5)P_5_; (23) DL-Ins(1,2,4,5,6)P_5_; (24) Ins(1,3,4,5,6)P_5_; (25) InsP_6_.
(TIFF)

Figure S4 **Proposed phytate degradation pathway (InsP_6_-InsP_4_) for** ***Ha*** **Phy and** ***Yk*** **Phy (a) and** ***Ec*** **Phy (b) at pH 4.0 based on HPIC identification of products.** Solid arrows indicate the preferred pathway, while hatched arrows indicate alternative routes. The numbers indicate the ratio of the observed isomers. *) DL-Ins(1,2,5,6)P_4_ and DL-Ins(2,3,4,5)P_4_ are stereoisomers and cannot be distinguished by HPIC.
(TIFF)

Figure S5 **The second, non-catalytic, MIHS binding site.** (a) Ribbon representation of three symmetry-related molecules in green, yellow and cyan with the phytate molecules shown in cylinders. (b) Stereo close-up. The model is shown in ball and stick, with the electron density for the ligand at the 1σ level. The residues belonging to different molecules are in the same colours as the corresponding molecules in (a). Figures S5 was drawn using CCP4mg [3].
(TIFF)

Figure S6 **Ribbon representation of the** ***Yk*** **Phy overall fold.** The α domain (residues 25–45 and 137–264) is shown in grey, the α/β domain is in blue. The four disulphide bridges are in sphere format and lie in surface loops. The orientation is similar to that of *Ha*Phy in Figure 5a of the main text.
(TIFF)

Supporting Information S1. (DOCX)
