# Supplementary figures and images for: Degradation of Phytate by the 6-Phytase from Hafnia alvei: A Combined Structural and Solution Study
Source: PLoS One. 2013 May 31;8(5):e65062. doi: 10.1371/journal.pone.0065062 (PMC3669009; doi:10.1371/journal.pone.0065062)

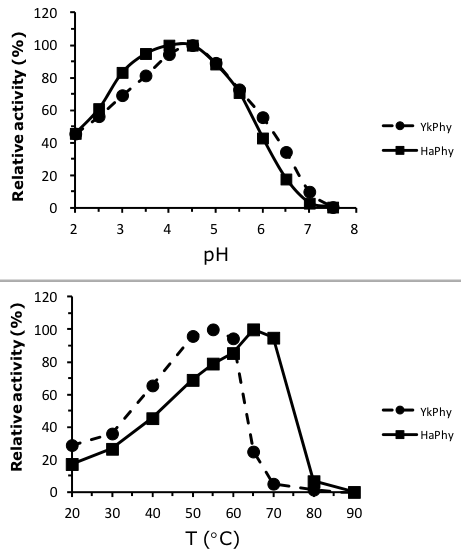

Supplement: Figure S1 — (a) The relative activity of the two enzymes as a function of pH (b) The relative activity as a function of temperature. In both a) and b) the values are relative % activity normalized to the value at optimum for each phytase. (TIFF) [file pone.0065062.s001.tiff]

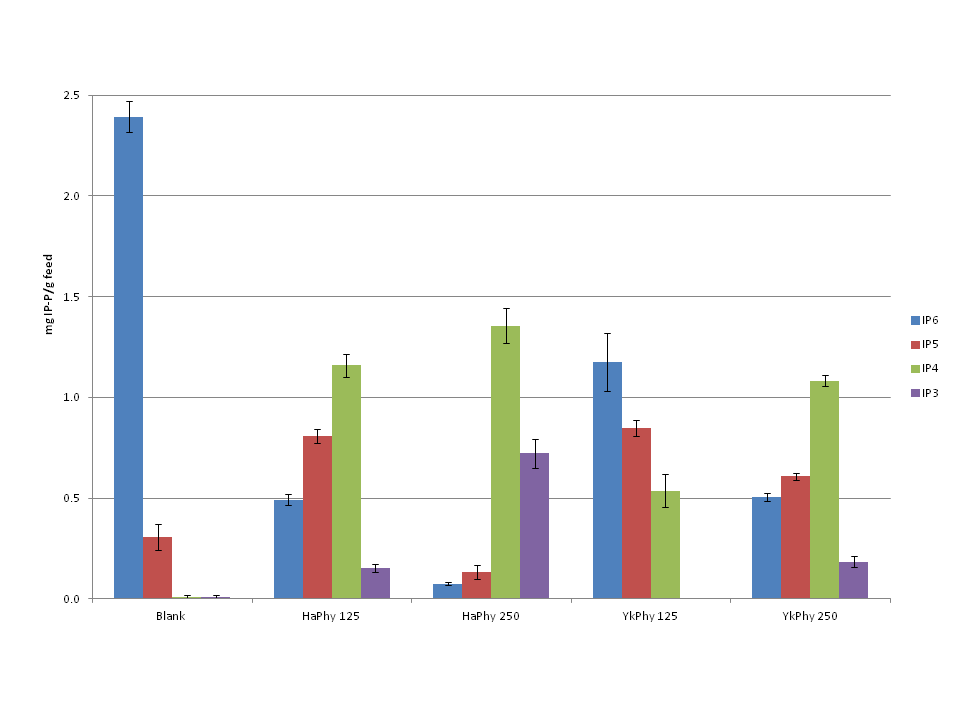

Supplement: Figure S2 — Residual inositol phosphates (InsP6-InsP3; mg InsP-P/g feed) after in vitro incubation without phytase or with Ha Phy or Yk Phy dosed at 125 and 250 FYT/kg feed. (TIFF) [file pone.0065062.s002.tiff]

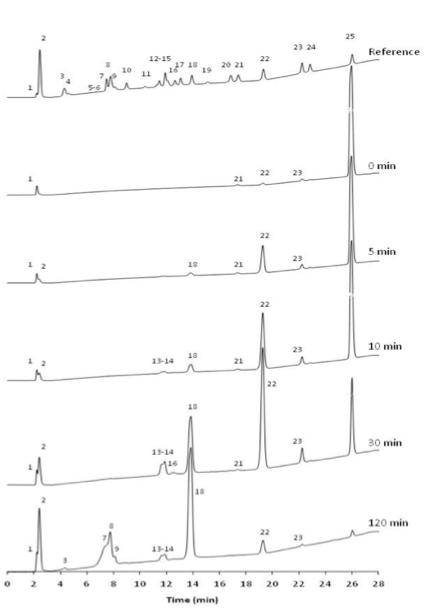

Supplement: Figure S3 — HPIC analysis of the hydrolysis products of myo-inositol hexakisphosphate (InsP6-InsP2) by the purified phytase after in vitro incubation for 0, 5, 10, 30 and 120 min at pH 4.0. Reference sample of hydrolysed Na-phytate. Peaks: (1) InsP1; (2) Phosphate; (3–4) InsP2; (5) Ins(1,3,5)P3; (6) Ins(2,4,6)P3; (7) DL-Ins(1,2,4)P3; (8) DL-Ins(1,2,6)P3, Ins(1,2,3)P3; (9) DL-Ins(1,4,5)P3; (10) DL-Ins(1,5,6)P3; (11)Ins(4,5,6)P3; (12) Ins(1,2,3,5)P4; (13) DL-Ins(1,2,4,6)P4; (14) DL-Ins(1,2,3,4)P3; (15) Ins(1,3,4,6)P4; (16) DL-Ins(1,2,4,5)P4; (17) DL-Ins(1,3,4,5)P4; (18) DL-Ins(1,2,5,6)P4; (19) Ins(2,4,5,6)P4; (20) DL-Ins(1,4,5,6)P4; (21) Ins(1,2,3,4,6)P5; (22) DL-Ins(1,2,3,4,5)P5; (23) DL-Ins(1,2,4,5,6)P5; (24) Ins(1,3,4,5,6)P5; (25) InsP6. (TIFF) [file pone.0065062.s003.tiff]

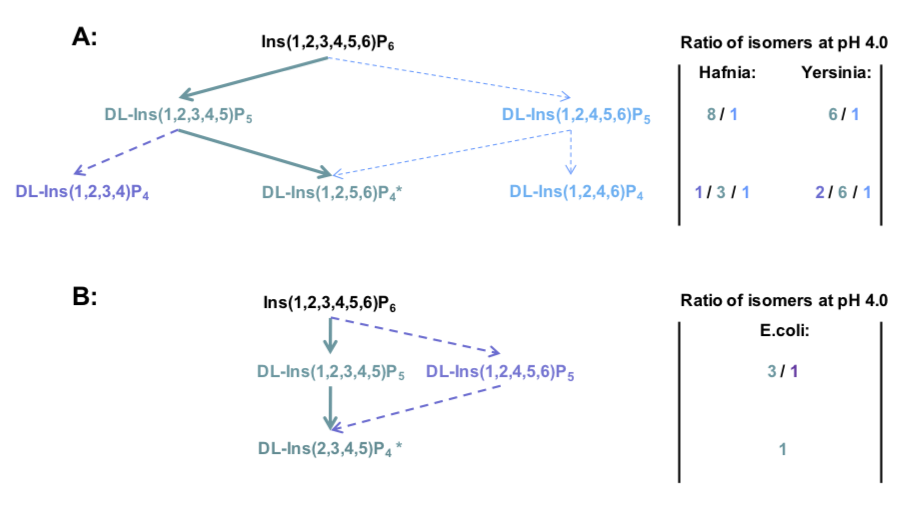

Supplement: Figure S4 — Proposed phytate degradation pathway (InsP6-InsP4) for Ha Phy and Yk Phy (a) and Ec Phy (b) at pH 4.0 based on HPIC identification of products. Solid arrows indicate the preferred pathway, while hatched arrows indicate alternative routes. The numbers indicate the ratio of the observed isomers. *) DL-Ins(1,2,5,6)P4 and DL-Ins(2,3,4,5)P4 are stereoisomers and cannot be distinguished by HPIC. (TIFF) [file pone.0065062.s004.tiff]

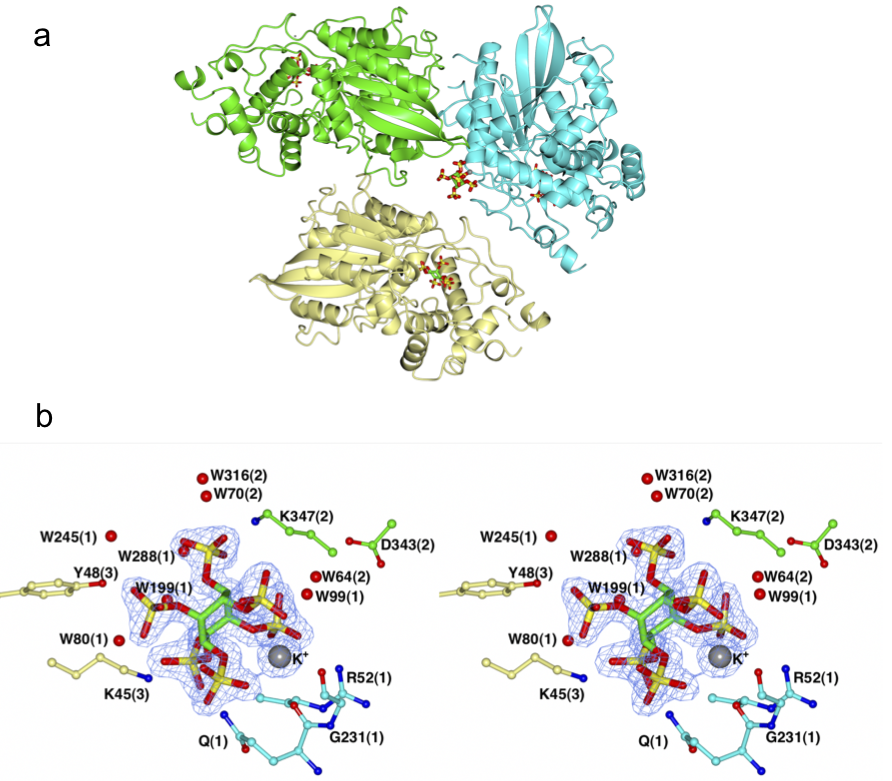

Supplement: Figure S5 — The second, non-catalytic, MIHS binding site. (a) Ribbon representation of three symmetry-related molecules in green, yellow and cyan with the phytate molecules shown in cylinders. (b) Stereo close-up. The model is shown in ball and stick, with the electron density for the ligand at the 1σ level. The residues belonging to different molecules are in the same colours as the corresponding molecules in (a). Figures S5 was drawn using CCP4mg [3]. (TIFF) [file pone.0065062.s005.tiff]

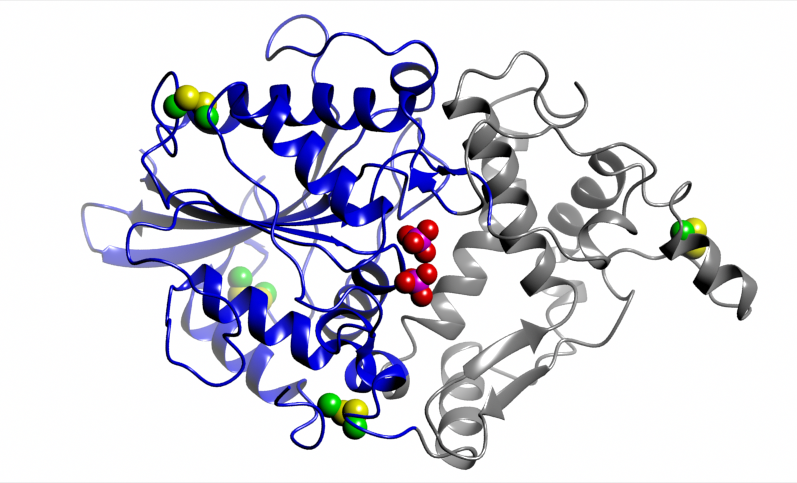

Supplement: Figure S6 — Ribbon representation of the Yk Phy overall fold. The α domain (residues 25–45 and 137–264) is shown in grey, the α/β domain is in blue. The four disulphide bridges are in sphere format and lie in surface loops. The orientation is similar to that of HaPhy in Figure 5a of the main text. (TIFF) [file pone.0065062.s006.tiff]
