# Supplementary material for: Degradation of Phytate by the 6-Phytase from Hafnia alvei: A Combined Structural and Solution Study
Source: PLoS One. 2013 May 31;8(5):e65062. doi: 10.1371/journal.pone.0065062 (PMC3669009; doi:10.1371/journal.pone.0065062)
Supplement: Supporting Information S1. — (DOCX) [file pone.0065062.s007.docx]

**Running title:** *The 6-phytase from Hafnia alvei ligand complex*

# Supporting information methods

***Cloning and expression.*** A multiple alignment was made of the following HAPs: *E. coli* phytase (SPTREMBL:Q8GN88), *Citrobacter gillenii* DSM 13694 phytase (geneseqp:aeh04533), *C. amalonaticus* ATCC 25407 phytase (geneseqp:aeh04535), *C. braakii* phytase (geneseqp:aeh04827), and ypo1648 *Y. pestis* CO92 (SPTREMBL:Q8ZFP6). Two degenerate oligonucleotide primers were designed on the basis of the consensus sequences:

2123fw: 5´- CATGGTGTGCGNGCNCCNACNAA -3´

2065rev: 5´- CCCACCAGGNGGNGTRTTRTCNGGYTG -3´

where Y designates T or C, R designates A or G, and N designates A, C, G or T. The primers were used for PCR screening of a number of bacterial species at annealing temperatures between 40 and 50°C. A partial phytase gene in the form of an approximately 950 bp PCR fragment was identified in *H. alvei* (DSM 19197) and *Y. kristensenii* (NN20123). The PCR fragments were isolated from agarose gel and the two fragments were sequenced using the same PCR primers from which the fragment was generated. By translation of the nucleotide sequence, it was confirmed that both DNA fragments were part of a HAP phytase gene. To obtain the full-length nucleotide sequence of the two genes, the DNA WALKING SPEEDUP™ Kit (DWSK-V102 from Seegene, Inc., Korea) was used, according to manufacturer’s instructions.

A 27 amino-acid signal peptide encoding polynucleotide of a native protease, Savinase™, from *Bacillus licheniformis* was fused by PCR in frame to the genes encoding the mature phytases. The DNA coding for the fusion polypeptides was integrated by homologous recombination into the chromosome of *B. subtilis* MB1053. The gene constructs were expressed under the control of a triple promoter system (as described in WO 99/43835), consisting of the promoters from the *B. licheniformis* α-amylase gene (amyL and amyQ), and the *B. thuringiensis* cryIIIA promoter including the mRNA stabilizing sequence. The gene coding for Chloramphenicol acetyl-transferase was used as marker, as described in [[1](#_ENREF_1)].Chloramphenicol resistant transformants were cultured in PS-1 medium (10% sucrose, 4% soybean flour, 1% Na_3_PO_4_·12H_2_O, 0.5% CaCO_3_, 0.01% pluronic acid) shaken at 250 RPM at 30°C. After 2-5 days incubation the supernatant was removed and the phytase activity was identified by applying 20 μl of the supernatant into 4 mm diameter holes punched out in 1% LSB-agarose plates containing 0.1 M sodium acetate pH 4.5 and 0.1% (w/v) phytate. The plates were left overnight at 37°C and a buffer consisting of 0.25 M CaCl_2_ , 500 mM MES pH 6.5 was poured over them. The plates were left at room temperature for 1h and the inositol-phosphate phosphatase/phytase activity was then identified as a clear zone.

Several phytase positive transformants from each of the two constructs were validated by DNA sequencing. One correct clone was selected from each construct and cultivated at 30°C and with 250 rpm for 6 days in SK-1M medium: 40 g sodium caseinate (Miprodan 30 from Arla), 200 g maltodextrin 01 (Glucidex 6, catalogue no. 332203 from Roquette), 50 g Soybean Meal, 0.1 ml Dowfax 63N10 (a non-ionic surfactant from Dow), tap water up to 1000 ml and CaCO_3_ tablet 0.5g/100 ml.

***Nucleotide sequence accession numbers.*** The full length nucleotide sequences encoding the phytase from *H. alvei* DSM 19197 and *Y. kristensenii* NN20123 have been deposited at the EMBL database under the accession numbers JQ394762 and JQ394763, respectively.

***Expression of the T308A HaPhy variant in Aspergillus oryzae*.** The construct comprising the HaPhy variant gene was used to construct expression vectors for *A. oryzae*, consisting of an expression cassette based on the *A. niger* neutral amylase II promoter fused to the *A. nidulans* triose phosphate isomerase non translated leader sequence (Pna2/tpi) and the *A. niger* amyloglycosidase terminator (Tamg). Also present on the plasmid was the *Aspergillus* selective marker amdS from *A. nidulans* enabling growth on acetamide as sole nitrogen source. The expression plasmids were transformed into *A. oryzae* as described by Lassen *et al*. [[2](#_ENREF_2)]. 10-20 strains for each construct were isolated, purified and cultivated in shake flasks.

***Purification.*** The fermentation supernatant with the phytase was centrifuged at 7200 rpm and 5°C for 1 h, filtered through a sandwich of four Whatman glass microfibre filters (2.7, 1.6, 1.2 and 0.7 μm) and finally passed through a sterile Seitz-EKS depth filter using pressure. The filtered supernatant was pre-treated by washing it with water and then concentrated using an ultrafiltration unit with a 10 kDa cut-off (Filtron Technology Corporation). Then pH was adjusted to 4.5 with 10% (w/v) acetic acid, which caused a minor precipitation. No activity was found in the precipitate and it was removed by filtration through a Fast PES bottle top filter with a 0.22 μm cut-off. After pre-treatment, the phytase was purified by chromatography on S Sepharose (approximately 50 ml in a XK26 column) using 50 mM sodium acetate pH 4.5 as buffer A and A + 1 M NaCl as buffer B. The fractions from the column were analysed for activity using *p*-nitrophenyl phosphate (Sigma, N-9389) as substrate and fractions with positive activity were pooled. Solid ammonium sulphate was added to the solution giving a final concentration of 1.5 M and the pH was adjusted to 6.0 using 6 M HCl. The phytase-containing solution was applied to a butyl-sepharose column (approximately 30 ml in a XK26 column) using 25 mM Bis-Tris pH 6.0, 1.5 M ammonium sulphate as buffer A and 25 mM Bis-Tris pH 6.0 as buffer B. The eluted fractions were analysed for phosphatase activity using *p*-nitrophenyl phosphate (Sigma, N-9389) as substrate. Finally, the solution containing the purified phytase was buffer-exchanged into 50 mM sodium acetate pH 4.5 and concentrated using an Amicon ultra-15 filtering device with a 30 kDa cut-off membrane. The molecular weight, as estimated from SDS-PAGE, was approximately 40 kDa and the purity was > 95%.

# *List of Uniprot entries for Figure 2 in the main text*

A7FH71: Y.pseudotuberculosis serotype O:1b (stra...

Q6CZF4: E.carotovora subsp. atroseptica (Pectoba...

O00092: N.fumigata (strain ATCC MYA-4609 / Af293...

O00093: E.nidulans (Aspergillus nidulans)

P08091: S.pombe (strain 972 / ATCC 24843) (Fissi...

Q01682: S.pombe (strain 972 / ATCC 24843) (Fissi...

P52289: K.lactis (strain ATCC 8585 / CBS 2359 / ...

P35842: S.cerevisiae (strain ATCC 204508 / S288c...

C4ZQA6: E.coli (strain K12 / MC4100 / BW2952)

Q676V7: C.freundii

Q6RK08: E.coli

Q6TAQ8: O.proteus

Q6U677: O.proteus

A6T657: K.pneumoniae subsp. pneumoniae (strain A...

F1XIJ0: E.coli O157:H7 str. 1044

Q32HS7: S.dysenteriae serotype 1 (strain Sd197)

A9R0K5: Y.pestis bv. Antiqua (strain Angola)

F3W045: S.boydii 3594-74

Q7UD08: S.flexneri

Q7WSY1: R.terrigena (Klebsiella terrigena)

A0JJX7: A.niger

Q84CN9: K.pneumoniae

A1CXB1: N.fischeri (strain ATCC 1020 / DSM 3700 ...

Q8GD20: P.syringae

Q8GN88: E.coli

A1X812: A.niger

A1XRK2: A.niger

A1XRK3: A.niger

Q8RKD6: E.coli

Q8RKD7: E.coli

A2QSK3: A.niger (strain CBS 513.88 / FGSC A1513)

Q0T656: S.flexneri serotype 5b (strain 8401)

E9TVX6: E.coli MS 60-1

Q8VQS2: K.pneumoniae

A2TBB4: S.capriottii

A2TEY4: A.niger

A2TEY5: A.oryzae

A2TEY6: Penicillium sp. Q7

A2TJJ4: A.ficuum

A3LUP9: S.stipitis (strain ATCC 58785 / CBS 6054...

A4QVW6: M.oryzae (strain 70-15 / ATCC MYA-4617 /...

A4REX0: M.oryzae (strain 70-15 / ATCC MYA-4617 /...

A1A9P8: E.coli O1:K1 / APEC

A5DRY8: L.elongisporus (strain ATCC 11503 / CBS ...

A5H2T5: L.elongisporus (strain ATCC 11503 / CBS ...

E3XYH5: S.flexneri 2a str. 2457T

O00085: A.terreus

O00100: A.terreus

P34753: A.awamori

P34752: A.niger

Q9C1T1: A.oryzae (strain ATCC 42149 / RIB 40)

Q0CLV1: A.terreus (strain NIH 2624 / FGSC A1156)

O00107: T.heterothallica (Myceliophthora thermop...

P34755: A.awamori

P34754: A.niger

A6RB65: A.capsulata (strain NAm1 / WU24) (Darlin...

P52291: P.pastoris (Yeast)

O60172: S.pombe (strain 972 / ATCC 24843) (Fissi...

P24031: S.cerevisiae (strain ATCC 204508 / S288c...

D3UEI8: S.cerevisiae (strain Lalvin EC1118 / Pri...

F4N9X5: S.bayanus (Yeast) (Saccharomyces uvarum)

B5VFP1: S.cerevisiae (strain AWRI1631) (Baker's ...

A6S3W2: B.fuckeliana (strain B05.10) (Noble rot ...

A6SFM5: B.fuckeliana (strain B05.10) (Noble rot ...

A6ZL39: S.cerevisiae (strain YJM789) (Baker's ye...

E7LRJ1: S.cerevisiae (strain VIN 13) (Baker's ye...

A6ZSK5: S.cerevisiae (strain YJM789) (Baker's ye...

A6ZXU6: S.cerevisiae (strain YJM789) (Baker's ye...

A7A091: S.cerevisiae (strain YJM789) (Baker's ye...

A7A095: S.cerevisiae (strain YJM789) (Baker's ye...

Q8CW75: E.coli O6

A7EX98: S.sclerotiorum (strain ATCC 18683 / 1980...

A7MF64: E.sakazakii (strain ATCC BAA-894)

C3TFD7: E.coli

A8Q3P1: M.globosa (strain ATCC MYA-4612 / CBS 79...

A8QAI9: M.globosa (strain ATCC MYA-4612 / CBS 79...

O00096: T.thermophilus

O74677: P.angusta (Yeast) (Hansenula polymorpha)

A2QIG7: A.niger (strain CBS 513.88 / FGSC A1513)

Q0CVC9: A.terreus (strain NIH 2624 / FGSC A1156)

Q0CZR1: A.terreus (strain NIH 2624 / FGSC A1156)

C4VUW0: E.coli BL21(DE3)

Q1DN23: C.immitis (Valley fever fungus)

Q5NRG9: Z.mobilis

A1JTE2: Y.enterocolitica serotype O:8 / biotype ...

Q1KNE3: A.oryzae

Q2H2W6: C.globosum (strain ATCC 6205 / CBS 148.5...

Q2MKJ5: A.niger

Q2U6N3: A.oryzae (strain ATCC 42149 / RIB 40)

Q2XQS0: A.niger

Q3HSD6: A.usamii

Q3LR77: A.awamori

Q4P6B3: U.maydis (strain 521 / FGSC 9021) (Smut ...

Q4P6I1: U.maydis (strain 521 / FGSC 9021) (Smut ...

Q55NU2: C.neoformans var. neoformans serotype D ...

Q59UY6: C.albicans (strain SC5314 / ATCC MYA-287...

Q59V13: C.albicans (strain SC5314 / ATCC MYA-287...

Q59V50: C.albicans (strain SC5314 / ATCC MYA-287...

Q5A947: C.albicans (strain SC5314 / ATCC MYA-287...

Q5AMR2: C.albicans (strain SC5314 / ATCC MYA-287...

Q5ASI5: E.nidulans (Aspergillus nidulans)

Q5GGT6: N.crassa

Q5KEM1: C.neoformans var. neoformans serotype D ...

Q5KEM2: C.neoformans var. neoformans serotype D ...

Q5XNQ8: A.niger

Q5XNQ9: N.fumigata (Aspergillus fumigatus)

Q6BM75: D.hansenii (strain ATCC 36239 / CBS 767 ...

Q6CCS5: Y.lipolytica (strain CLIB 122 / E 150) (...

Q6CSM6: K.lactis (strain ATCC 8585 / CBS 2359 / ...

Q6CYI2: K.lactis (strain ATCC 8585 / CBS 2359 / ...

Q6GYA8: A.niger

Q6J336: A.oryzae

Q6R519: A.niger

Q6T9Z6: A.niger

Q6YNE9: P.oxalicum

Q7S9V5: N.crassa (strain ATCC 24698 / 74-OR23-1A...

Q7SEH2: N.crassa (strain ATCC 24698 / 74-OR23-1A...

Q8J255: A.ficuum

Q8X1W7: M.purpureus (Monascus anka)

Q96VF5: T.pubescens (White-rot fungus)

Q96VH9: P.lycii

Q96VK8: cf. Ceriporia sp. CBS 100231

Q96VK9: cf. Ceriporia sp. CBS 100231

Q96VT0: A.pediades

Q9HEQ0: A.ficuum

Q9UUZ7: A.niger

Q9Y846: K.lactis (Yeast) (Candida sphaerica)

Q2VY22: C.braakii

Q000T0: Y.intermedia

Q003Y3: C.amalonaticus

E7T800: S.flexneri CDC 796-83

1DKN: ESCHERICHIA COLI

A2UIN2: E.coli B

A9XD02: A.niger

B0DV43: L.bicolor (strain S238N-H82 / ATCC MYA-4...

B0Y655: N.fumigata (strain CEA10 / CBS 144.89 / ...

Q0V0X4: P.nodorum (strain SN15 / ATCC MYA-4574 /...

B1ERR8: E.albertii TW07627

B1LJ17: E.coli (strain SMS-3-5 / SECEC)

B2KSJ7: Buttiauxella sp. GC21

B2AS87: P.anserina (strain S / ATCC MYA-4624 / D...

B2TTR5: S.boydii serotype 18 (strain CDC 3083-94...

B2WJV4: P.tritici-repentis (strain Pt-1C-BFP) (W...

B3HSZ0: E.coli F11

B3ICM2: E.coli E22

B3IMU8: E.coli E110019

B3LGS8: S.cerevisiae (strain RM11-1a) (Baker's y...

B3LN59: S.cerevisiae (strain RM11-1a) (Baker's y...

B4X9S4: Y.rohdei

B4XT20: D.dadantii

B4XT21: D.paradisiaca

B4XY38: P.carotovorum subsp. carotovorum (Erwini...

B3VPB2: A.japonicus

B3VPB3: A.niger

E7I3B1: E.coli E128010

B3WZG2: S.dysenteriae 1012

B5FV69: K.lactis (strain ATCC 8585 / CBS 2359 / ...

Q6BUR8: D.hansenii (strain ATCC 36239 / CBS 767 ...

B5VE53: S.cerevisiae (strain AWRI1631) (Baker's ...

B5VE54: S.cerevisiae (strain AWRI1631) (Baker's ...

B5VKE4: S.cerevisiae (strain AWRI1631) (Baker's ...

B5XZW1: K.pneumoniae (strain 342)

B6H7V5: P.chrysogenum (strain ATCC 28089 / DSM 1...

B6HVH7: P.chrysogenum (strain ATCC 28089 / DSM 1...

E8Y2S2: E.coli (strain ATCC 55124 / KO11)

B6JZD6: S.japonicus (strain yFS275 / FY16936) (F...

B6QGZ8: P.marneffei (strain ATCC 18224 / CBS 334...

B6QH60: P.marneffei (strain ATCC 18224 / CBS 334...

B6QLB5: P.marneffei (strain ATCC 18224 / CBS 334...

B6RGT0: Pectobacterium sp. S27

B6RGT1: Y.kristensenii

E3XUT5: E.coli 2362-75

B7LQH6: E.fergusonii (strain ATCC 35469 / DSM 13...

B7M8W1: E.coli O8 (strain IAI1)

E9V4V1: E.coli H252

B7MPS0: E.coli O81 (strain ED1a)

B7N3E3: E.coli O17:K52:H18 (strain UMN026 / ExPE...

B7NLD7: E.coli O7:K1 (strain IAI39 / ExPEC)

B8MHX6: T.stipitatus (strain ATCC 10500 / CBS 37...

B8MHX7: T.stipitatus (strain ATCC 10500 / CBS 37...

B8N9F8: A.flavus (strain ATCC 200026 / FGSC A112...

B8NKJ2: A.flavus (strain ATCC 200026 / FGSC A112...

B8P9R1: P.placenta (strain ATCC 44394 / Madison ...

B8PES4: P.placenta (strain ATCC 44394 / Madison ...

B8PES5: P.placenta (strain ATCC 44394 / Madison ...

B8QJH4: Y.frederiksenii

E4PCD8: E.coli O83:H1 (strain NRG 857C / AIEC)

B9VHW1: Citrobacter sp. Gc-8-a

B9WFQ7: C.dubliniensis (strain CD36 / ATCC MYA-6...

B9WL31: C.dubliniensis (strain CD36 / ATCC MYA-6...

B9WL50: C.dubliniensis (strain CD36 / ATCC MYA-6...

C0NMH2: A.capsulata (strain G186AR / H82 / ATCC ...

C1D6G9: L.hongkongensis (strain HLHK9)

C1F6S1: A.capsulatum (strain ATCC 51196 / DSM 11...

C1G7E8: P.brasiliensis (strain Pb18)

C3X2L1: O.formigenes HOxBLS

C3X9E8: O.formigenes OXCC13

C4JG72: U.reesii (strain UAMH 1704)

C4K8D0: H.defensa subsp. Acyrthosiphon pisum (st...

C5BDT1: E.ictaluri (strain 93-146)

C5DDE6: L.thermotolerans (strain ATCC 56472 / CB...

C5DFV5: L.thermotolerans (strain ATCC 56472 / CB...

C5FGG6: A.otae (strain ATCC MYA-4605 / CBS 11348...

F2TIR8: A.dermatitidis (strain ATCC 18188 / CBS ...

C4S4J5: Y.bercovieri ATCC 43970

C4S833: Y.mollaretii ATCC 43969

C4T757: Y.intermedia ATCC 29909

C4TZX7: Y.kristensenii ATCC 33638

C4U759: Y.aldovae ATCC 35236

C4UWA3: Y.rohdei ATCC 43380

C4X5B9: K.pneumoniae subsp. pneumoniae NTUH-K204...

C4Y8Q7: C.lusitaniae (strain ATCC 42720) (Yeast)...

C4YHN9: C.albicans (strain WO-1) (Yeast)

C4YL49: C.albicans (strain WO-1) (Yeast)

C4YL67: C.albicans (strain WO-1) (Yeast)

C5JDT4: A.dermatitidis (strain SLH14081) (Blasto...

C5M245: C.tropicalis (strain ATCC MYA-3404 / T1)...

C5M4A0: C.tropicalis (strain ATCC MYA-3404 / T1)...

C5M4B2: C.tropicalis (strain ATCC MYA-3404 / T1)...

C6H286: A.capsulata (strain H143) (Darling's dis...

C5PJN7: C.posadasii (strain C735) (Valley fever ...

C5TF24: Z.mobilis subsp. mobilis ATCC 10988

C6CQ55: D.zeae (strain Ech1591)

C6DHE0: P.carotovorum subsp. carotovorum (strain...

C6NFQ9: P.wasabiae WPP163

C7GW56: S.cerevisiae (strain JAY291) (Baker's ye...

C7ZPL0: N.haematococca (strain 77-13-4 / ATCC MY...

C7YH73: N.haematococca (strain 77-13-4 / ATCC MY...

E6W7W9: Pantoea sp. (strain At-9b)

C8QTR3: D.dadantii Ech586

C8V9Z6: A.nidulans FGSC A4

C8WD87: Z.mobilis subsp. mobilis (strain NCIB 11...

C8Z7M5: S.cerevisiae (strain Lalvin EC1118 / Pri...

C9Y2R9: C.turicensis (strain DSM 18703 / LMG 238...

3K4P: ASPERGILLUS NIGER

D0YJP3: K.variicola At-22

D0ZGA3: E.tarda (strain EIB202)

D1ZCK3: S.macrospora (strain ATCC MYA-333 / DSM ...

D1ZHA8: S.macrospora (strain ATCC MYA-333 / DSM ...

D2ABN7: S.flexneri serotype X (strain 2002017)

D2NGG5: E.coli O150:H5 (strain SE15)

D3H0K2: E.coli O44:H18 (strain 042 / EAEC)

D3UEI7: S.cerevisiae (strain Lalvin EC1118 / Pri...

D4ANW6: A.benhamiae (strain ATCC MYA-4681 / CBS ...

D4DAW7: T.verrucosum (strain HKI 0517)

D4DZK6: S.odorifera DSM 4582

D4F403: E.tarda ATCC 23685

D4GL80: P.ananatis (strain LMG 20103)

1DKL: ESCHERICHIA COLI

1IHP: ASPERGILLUS FICUUM

1QFX: ASPERGILLUS NIGER

1SK9: ASPERGILLUS FUMIGATUS

2GFI: DEBARYOMYCES CASTELLII

2WNH: KLEBSIELLA PNEUMONIAE

D5G7S5: T.melanosporum (strain Mel28) (Perigord ...

D5HQ11: A.ficuum

E1HGN8: E.coli MS 146-1

D6I8P6: E.coli B185

D6IM53: E.coli FVEC1412

D6J8U6: E.coli B354

D6UN52: Acidobacterium sp. MP5ACTX8

F8NFX0: S.lacrymans var. lacrymans (strain S7.9)...

F8NQK5: S.lacrymans var. lacrymans (strain S7.9)...

F8P7P1: S.lacrymans var. lacrymans (strain S7.9)...

F8P7P2: S.lacrymans var. lacrymans (strain S7.9)...

D9D7K9: Y.enterocolitica

D9I0I9: P.oxalicum

D7ZG57: E.coli MS 69-1

E1IQ00: E.coli MS 145-7

D8A7S4: E.coli MS 21-1

D8CBX2: E.coli MS 185-1

D8ESZ3: E.coli MS 107-1

D9Y5P2: B.bacterium 1_1_47

D9YEY3: Desulfovibrio sp. 3_1_syn3

D8QCL9: S.commune (strain H4-8 / FGSC 9210) (Spl...

E0QXF6: E.coli NC101

E0SEL3: D.dadantii (strain 3937) (Erwinia chrysa...

D7X269: E.coli MS 198-1

D7XJ18: E.coli MS 84-1

E1I137: E.coli MS 78-1

E0LTP4: Pantoea sp. aB

E2DQG8: Aspergillus sp. A25

E1SF61: P.vagans (strain C9-1) (Pantoea agglomer...

E3RDI6: P.teres f. teres (strain 0-1) (Barley ne...

E3UHI1: A.niger

E3UHI2: A.niger

E3UHI3: A.niger

E3UHI4: A.niger

E3UHI5: A.niger

E3UHI6: A.niger

E3UHI7: A.niger

E3KMU2: Puccinia graminis f. sp. tritici (strain...

E3L424: Puccinia graminis f. sp. tritici (strain...

E3QEG3: C.graminicola (strain M1.001 / M2 / FGSC...

E3QJC2: C.graminicola (strain M1.001 / M2 / FGSC...

E4V5L2: A.gypseum (strain ATCC MYA-4604 / CBS 11...

E4ZRX9: L.maculans (strain JN3 / isolate v23.1.3...

E5Y8B1: B.wadsworthia 3_1_6

E5YEV0: E.bacterium 9_2_54FAA

E6AVJ3: E.coli MS 16-3

E6AZM9: E.coli 3431

E6R8S2: C.gattii serotype B (strain WM276 / ATCC...

E6ZNK6: S.reilianum (strain SRZ2) (Maize head sm...

E6ZPL0: S.reilianum (strain SRZ2) (Maize head sm...

E7B4V4: Y.enterocolitica subsp. palearctica sero...

F9X862: M.graminicola (strain CBS 115943 / IPO32...

F4RDC5: M.larici-populina (strain 98AG31 / patho...

F4S1C3: M.larici-populina (strain 98AG31 / patho...

F4SC54: M.larici-populina (strain 98AG31 / patho...

E9DZF4: M.acridum (strain CQMa 102)

E9EDF3: M.acridum (strain CQMa 102)

E9EMM2: M.robertsii (strain ARSEF 23 / ATCC MYA-...

E9EVY2: M.robertsii (strain ARSEF 23 / ATCC MYA-...

E7NEV8: S.cerevisiae (strain FostersO) (Baker's ...

E7NPM6: S.cerevisiae (strain FostersO) (Baker's ...

E7QAV4: S.cerevisiae (strain FostersB) (Baker's ...

E7QBX3: S.cerevisiae (strain Zymaflore VL3) (Bak...

E7QCM0: S.cerevisiae (strain Zymaflore VL3) (Bak...

E7R2R7: P.angusta (strain ATCC 26012 / NRRL Y-75...

E7R3S7: P.angusta (strain ATCC 26012 / NRRL Y-75...

E9KPK6: uncultured organism

E9M258: A.niger

E8LI73: S.hippei YIT 12066

E9TEJ1: E.coli MS 117-3

E9U5U5: E.coli MS 57-2

E9WD37: E.coli E1520

E9XMY2: E.coli TW10509

E9YSK1: E.coli M863

E9Z301: E.fergusonii B253

E9DES7: C.posadasii (strain RMSCC 757 / Silveira...

F0XF16: G.clavigera (strain kw1407 / UAMH 11150)...

F0JRC5: E.fergusonii ECD227

F4N6H4: Y.enterocolitica W22703

E7SJP5: S.dysenteriae CDC 74-1112

E7U3X0: E.coli WV_060327

F0G7E1: Burkholderia sp. TJI49

E7JGQ2: E.coli RN587/1

E7K9V4: S.cerevisiae (strain AWRI796) (Baker's y...

E7LRJ0: S.cerevisiae (strain VIN 13) (Baker's ye...

E7LSF7: S.cerevisiae (strain VIN 13) (Baker's ye...

F2RS84: T.tonsurans (strain CBS 112818) (Scalp r...

F1Z9L7: N.nitrogenifigens DSM 19370

F2Q1B8: T.equinum (strain ATCC MYA-4606 / CBS 12...

F2ENL5: P.ananatis (strain AJ13355)

F2SSQ2: T.rubrum (strain ATCC MYA-4607 / CBS 118...

F2VRZ7: E.agglomerans (Erwinia herbicola) (Panto...

F2VRZ8: Pseudomonas sp. 206

F2VRZ9: Pseudomonas sp. 206

F4VTB9: E.coli H299

F4ZNF9: Z.mays (Maize)

F3Q7Y1: Klebsiella sp. MS 92-3

F3QIC1: P.excrementihominis YIT 11859

F3WGT7: S.boydii 5216-82

F5M6X4: E.coli AA86

F5MJU5: S.flexneri K-218

F5NEJ8: S.flexneri K-272

F6IQ86: Shigella sp. CD2

F4SX62: E.coli M605

F4TCL1: E.coli M718

F4U798: E.coli TA143

F4V0T0: E.coli TA280

F5VK97: C.sakazakii E899

F7R6Q1: S.flexneri J1713

F7VY10: S.macrospora (strain ATCC MYA-333 / DSM ...

F7MV46: E.coli PCN033

F8MFE1: N.tetrasperma (strain FGSC 2508 / ATCC M...

F8N344: N.tetrasperma (strain FGSC 2508 / ATCC M...

F9FW73: F.oxysporum Fo5176

F9FXR8: F.oxysporum Fo5176

G0D5I8: E.coli NA114

G0E052: E.aerogenes (Aerobacter aerogenes)

G0GT22: K.pneumoniae

# Supporting information figure legends

**Figure S1.** (a) The relative activity of the two enzymes as a function of pH (b) The relative activity as a function of temperature. In both a) and b) the values are relative % activity normalized to the value at optimum for each phytase.

**Figure S2**. Residual inositol phosphates (InsP_6_-InsP_3_; mg InsP-P/g feed) after *in vitro* incubation without phytase or with *Ha*Phy or *Yk*Phy dosed at 125 and 250 FYT/kg feed.

**Figure S3.** HPIC analysis of the hydrolysis products of myo-inositol hexakisphosphate (InsP_6_-InsP_2_) by the purified phytase after *in vitro* incubation for 0, 5, 10, 30 and 120 min at pH 4.0. Reference sample of hydrolysed Na-phytate. Peaks: (1) InsP_1_; (2) Phosphate; (3-4) InsP_2_; (5) Ins(1,3,5)P_3_; (6) Ins(2,4,6)P_3_; (7) DL-Ins(1,2,4)P_3_; (8) DL-Ins(1,2,6)P_3_, Ins(1,2,3)P_3_; (9) DL-Ins(1,4,5)P_3_; (10) DL-Ins(1,5,6)P_3_; (11)Ins(4,5,6)P_3_; (12) Ins(1,2,3,5)P_4_; (13) DL-Ins(1,2,4,6)P_4_; (14) DL-Ins(1,2,3,4)P_3_; (15) Ins(1,3,4,6)P_4_; (16) DL-Ins(1,2,4,5)P_4_; (17) DL-Ins(1,3,4,5)P_4_; (18) DL-Ins(1,2,5,6)P_4_; (19) Ins(2,4,5,6)P_4_; (20) DL-Ins(1,4,5,6)P_4_; (21) Ins(1,2,3,4,6)P_5_; (22) DL-Ins(1,2,3,4,5)P_5_; (23) DL-Ins(1,2,4,5,6)P_5_; (24) Ins(1,3,4,5,6)P_5_; (25) InsP_6_.

**Figure S4**. Proposed phytate degradation pathway (InsP_6_-InsP_4_) for *Ha*Phy and *Yk*Phy (a) and *Ec*Phy (b) at pH 4.0 based on HPIC identification of products. Solid arrows indicate the preferred pathway, while hatched arrows indicate alternative routes. The numbers indicate the ratio of the observed isomers. *) DL-Ins(1,2,5,6)P_4_ and DL-Ins(2,3,4,5)P_4_ are stereoisomers and cannot be distinguished by HPIC.

**Figure S5**. The second, non-catalytic, MIHS binding site. (a) Ribbon representation of three symmetry-related molecules in green, yellow and cyan with the phytate molecules shown in cylinders. (b) Stereo close-up. The model is shown in ball and stick, with the electron density for the ligand at the 1σ level. The residues belonging to different molecules are in the same colours as the corresponding molecules in (a). Figures S5 was drawn using CCP4mg [[3](#_ENREF_3)]

**Figure S6**. Ribbon representation of the *Yk*Phy overall fold. The α domain (residues 25-45 and 137-264) is shown in grey, the α/β domain is in blue. The four disulphide bridges are in sphere format and lie in surface loops. The orientation is similar to that of *Ha*Phy in Figure 5a of the main text.

# Supporting information references

# Diderichsen B, Poulsen GB, Jorgensen ST (1993) A useful cloning vector for Bacillus subtilis. Plasmid 30: 312-315.

# Lassen SF, Breinholt J, Ostergaard PR, Brugger R, Bischoff A, et al. (2001) Expression, gene cloning, and characterization of five novel phytases from four basidiomycete fungi: Peniophora lycii, Agrocybe pediades, a Ceriporia sp., and Trametes pubescens. Applied and environmental microbiology 67: 4701-4707.

# McNicholas S, Potterton E, Wilson KS, Noble ME (2011) Presenting your structures: the CCP4mg molecular-graphics software. Acta crystallographica Section D, Biological crystallography 67: 386-394.
